# Construction and validation of an angiogenesis-related lncRNA prognostic model in lung adenocarcinoma

**DOI:** 10.3389/fgene.2023.1083593

**Published:** 2023-03-14

**Authors:** Quan Gong, Xianda Huang, Xiaobo Chen, Lijuan Zhang, Chunyan Zhou, Shijuan Li, Tingting Song, Li Zhuang

**Affiliations:** ^1^ Department of Palliative Medicine, The Third Affiliated Hospital of Kunming Medical University, Yunnan Cancer Hospital, Kunming, Yunnan, China; ^2^ Emergency Department, The Third Affiliated Hospital of Kunming Medical University, Yunnan Cancer Hospital, Kunming, Yunnan, China; ^3^ Department of Thoracic Surgery, The Third Affiliated Hospital of Kunming Medical University, Yunnan Cancer Hospital, Kunming, Yunnan, China

**Keywords:** angiogenesis, long non-coding RNAs (lncRNAs), lung adenocarcinoma (LUAD), prognosis, ceRNA, tumor mutation burden

## Abstract

**Background:** There is increasing evidence that long non-coding RNAs (lncRNAs) can be used as potential prognostic factors for cancer. This study aimed to develop a prognostic model for lung adenocarcinoma (LUAD) using angiogenesis-related long non-coding RNAs (lncRNAs) as potential prognostic factors.

**Methods:** Transcriptome data from The Cancer Genome Atlas (TCGA) and Gene Expression Omnibus (GEO) were analyzed to identify aberrantly expressed angiogenesis-related lncRNAs in LUAD. A prognostic signature was constructed using differential expression analysis, overlap analysis, Pearson correlation analysis, and Cox regression analysis. The model’s validity was assessed using K-M and ROC curves, and independent external validation was performed in the GSE30219 dataset. Prognostic lncRNA-microRNA (miRNA)-messenger RNA (mRNA) competing endogenous RNA (ceRNA) networks were identified. Immune cell infiltration and mutational characteristics were also analyzed. The expression of four human angiogenesis-associated lncRNAs was quantified using quantitative real-time PCR (qRT-PCR) gene arrays.

**Results:** A total of 26 aberrantly expressed angiogenesis-related lncRNAs in LUAD were identified, and a Cox risk model based on LINC00857, RBPMS-AS1, SYNPR-AS1, and LINC00460 was constructed, which may be an independent prognostic predictor for LUAD. The low-risk group had a significant better prognosis and was associated with a higher abundance of resting immune cells and a lower expression of immune checkpoint molecules. Moreover, 105 ceRNA mechanisms were predicted based on the four prognostic lncRNAs. qRT-PCR results showed that LINC00857, SYNPR-AS1, and LINC00460 were significantly highly expressed in tumor tissues, while RBPMS-AS1 was highly expressed in paracancerous tissues.

**Conclusion:** The four angiogenesis-related lncRNAs identified in this study could serve as a promising prognostic biomarker for LUAD patients.

## 1 Introduction

Nowadays, malignant tumors are posing a grave danger to human health. Lung cancer, which accounts for 11.6% of total cancer cases, is the most frequent form of cancer and the leading cause of death (18.4% of total cancer deaths) (Bray et al., 2018). Lung adenocarcinoma (LUAD) stands as the principal histologic subtype of lung cancer (Ferlay et al., 2018; Cao et al., 2020; Xu et al., 2020), with a 5-year survival rate of approximately 15% (Zhang et al., 2019; Jurisic et al., 2020). Unfortunately, the vast majority of patients with LUAD are diagnosed at an advanced stage, which contributes to the low survival rate. Currently, there is a lack of biomarkers for predicting poor prognosis and treatment prediction in LUAD patients. It is urgent to identify novel biomarkers to identify high-risk patients with poor prognosis for early diagnosis and intensive treatment regimens to improve their outcomes. Tumor angiogenesis plays an important role not only in tumor growth and progression (Folkman, 2002), but also in tumor invasion and metastasis (Xiaoxia et al., 2020). However, the mechanisms associated between LUAD and angiogenesis requires further investigation.

Tumor endothelial cell-derived cadherin-2, which promotes angiogenesis, is prognostically significant in lung adenocarcinoma (Zhuo et al., 2019). Numerous studies have attempted to inhibit tumor angiogenesis to control the development of lung adenocarcinoma (Chen et al., 2019; Wan et al., 2021; Li and Lu, 2022), indicating the research value of angiogenesis-related genes in the prognosis of lung cancer patients. In addition, Studies have shown that long non-coding RNAs (lncRNAs) have been playing an important role in cancer suppression. lncRNAs are involved in various biological processes of cancer, including lung cancer, such as proliferation, invasion, and metastasis. Recent evidence suggests that lncRNAs are involved in TKI resistance in NSCLC, especially linear plasticity-mediated resistance (Dong et al., 2021). In addition, long non-coding RNAs are implicated in the tumor microenvironment of lung cancer (Dai et al., 2021). For instance, Cong et al. found that in lung adenocarcinoma, depletion of lncRNA linc00665 could promote angiogenesis in lung inflammatory carcinoma (Cong et al., 2020). However, the relationship between angiogenesis-related lncRNAs and LUAD prognosis remains unclear and more research is needed.

This study aims to investigate the impact mechanism of angiogenesis-related lncRNAs on the prognosis of lung adenocarcinoma. Multiple angiogenesis-related lncRNAs were used to construct prognostic features by various analytical methods in an attempt to elucidate the relationship between angiogenesis-related lncRNAs and the prognosis and progression of LUAD.

## 2 Materials and methods

### 2.1 Data source

We obtained transcriptome data (lncRNA and messenger RNA (mRNA) sequencing data) and clinical data from the TCGA database for 579 samples, containing 521 LUAD samples and 58 normal samples. The 491 LUAD samples with complete survival information and clinical information were used for model construction and evaluation analysis. Meanwhile, we obtained the GSE31210 dataset (https://www.ncbi.nlm.nih.gov/geo/query/acc.cgi?acc=GSE31210) (Okayama et al., 2012; Yamauchi et al., 2012) and GSE30219 dataset (https://www.ncbi.nlm.nih.gov/geo/query/acc.cgi?acc=GSE30219) (Rousseaux et al., 2013) from the GEO database. The GSE31210 dataset contained lncRNA sequencing data from 20 normal samples and 226 LUAD samples. The GSE30219 dataset contained 293 LUAD samples that were included for independent external validation analysis of the prognostic signature.

### 2.2 Acquisition of the angiogenesis-related genes (ARGs)

Using Angiogenesis as the keyword, we searched through the Gene Cards online database (https://www.genecards.org/) to select genes with a score >5 and belonging to Protein Coding as ARGs, and we obtained a total of 104 ARGs (Supplementary Table S1).

### 2.3 Differential expression analysis

To identify differentially expressed genes (DEGs) in the TCGA database and GSE31210 dataset, the study performed a differential expression analysis using R package limma (Smyth, 2005). The differential comparison scheme was LUAD versus (vs.) normal. Genes that satisfied |log_2_ fold change (FC) > 0.5 and *p* < 0.05 between LUAD and normal samples were identified as differentially expressed genes (DEGs).

### 2.4 Overlap analysis

Overlap analysis was used to obtain differentially expressed lncRNAs (DE-lncRNAs) that were commonly found in LUAD. To ensure consistent expression levels of lncRNAs, we performed overlap analysis on the up- and downregulated lncRNAs identified in the TCGA and GES31210 datasets, respectively. The overlap analysis was performed in the Jvenn online tool (http://jvenn.toulouse.inra.fr/app/example.html), which can output a Venn diagram.

### 2.5 Pearson correlation analysis

Pearson correlation analysis is a statistical method that measures the linear relationship between two continuous variables. In this case, it was use to identify differentially expressed ARG-related lncRNAs in LUAD (LUAD-related DE-lncRNAs). Overlapping DE-lncRNAs with any of the identified differentially expressed ARGs (DE-ARGs) satisfying |cor| > 0.4 and *p* < 0.001 would be referred to as LUAD-related DE-lncRNAs for the subsequent analysis.

### 2.6 Construction, evaluation, and validation of the Cox risk model

We extracted the expression profiles and clinical information of 491 LUAD samples contained in the TCGA-LUAD database. The dataset was divided into a TCGA-training set (*n* = 343) and a TCGA-test set (*n* = 148) at random, with a 7:3 ratio. The TCGA-training set was used for screening prognostic lncRNAs, constructing a prognostic signature, and evaluating efficacy, while the TCGA-test set was utilized for internal validation of the prognostic signature. Additionally, the GSE30219 dataset from the GEO database was employed for independent external validation analysis of the prognostic signature. Based on the TCGA-training set, Cox regression analysis was employed to screen variables associated with LUAD prognosis and construct a prognostic signature of lncRNAs. Briefly, obtained LUAD-related DE-lncRNAs were included in the univariate Cox regression analysis. According to *p* < 0.05, variables meeting the conditions were enrolled in multivariate Cox analysis with stepwise regression. Variables generated by multivariate Cox analysis were regarded as the optimal lncRNAs for constructing the prognostic signature. In the Cox regression analysis, variables with a hazard ratio (HR) > 1 were deemed risk factors/oncogenes, while those with HR < 1 were regarded as protective factors. The prognostic predictive validity of the prognostic signature of lncRNAs was assessed by a risk scoring system, whereby the risk score for each sample was calculated based on the expression value (ecpr) of prognostic lncRNAs in that sample and the regression coefficient (coef) of prognostic lncRNAs generated by multivariate Cox analysis. The risk score was calculated as follows:

$$risk\;score=\sum_{i=1}^{n}coef\left({gene}_{i}\right)\times expr\left({gene}_{i}\right)$$

The coef and expr represent the regression coefficient and expression of each lncRNA, respectively. The risk scores were subject to computation performed in three datasets (TCGA-training set, TCGA-test set, and GSE30219 dataset). The LUAD samples in each dataset were classified into high- and low-risk groups according to the median value of the risk score in the corresponding dataset. The difference in overall survival (OS) between the two risk subgroups was analyzed by K-M curves. ROC curves were plotted and AUC values were calculated to assess the accuracy and specificity of the risk score in predicting patient OS.

### 2.7 Independent prognostic value analysis and construction of a nomogram

Clinical characteristics, including age, gender, stage, and TNM stage, as well as risk score, were included in the univariate Cox regression analysis. The significance threshold was set at *p* < 0.05. The variables obtained by univariate Cox analysis were further entered into multivariate Cox analysis, and finally, the variables with *p* < 0.05 were considered independent prognostic factors for LUAD. In addition, we divided LUAD patients into distinct subgroups of clinical characteristics and analyzed the levels of risk scores in the different subgroups using the Wilcoxon rank-sum test. The clinical subgroups were classified as follows: age subgroup (≤66 and >66 groups), sex subgroup (male and female groups), stage subgroup (stage I, stage II, stage III, and stage IV groups), tumor primary status (T stage) subgroup (T1, T2, T3, and T4 groups), lymph node involvement (N stage) subgroup (N0, N1, N2, and N3 groups), and distant metastasis status (M stage) subgroup (M0 and M1 subgroups). The detected independent prognostic factors were enrolled into establishing a nomogram by using rms package to predict the survival probability of LUAD patients (Iasonos et al., 2008). The calibration curves were plotted to assess the prediction ability, and the clinical net benefit was estimated by Decision curve analysis (DCA) (Vickers et al., 2008; Chi et al., 2022).

### 2.8 Construction of competing endogenous RNA (ceRNA) network

To initially explore the regulatory mechanisms of prognostic lncRNAs, we predicted the prognostic lncRNA-microRNA (miRNA) and miRNA-mRNA interactions through multiple databases, including DIANA-tools (www.microrna.gr) (Vlachos and Hatzigeorgiou, 2017), miRWalk (http://mirwalk.uni-hd.de/) (Sticht et al., 2018), miRDB (http://mirdb.org) (Chen and Wang, 2020), miRTarBase (http://miRTarBase.cuhk.edu.cn/) (Huang et al., 2020), and TargetScan (http://www.targetscan.org) (Huang et al., 2020) databases. Briefly, we predicted the miRNAs targeted by prognostic lncRNAs using the LncBase Predicted v.2 database of the DIANA online tool. Then, the mRNAs targeted by the above miRNAs were predicted by the miRWalk database. Next, we uploaded the list of obtained mRNAs to miRDB, miRTarBase, and TargetScan databases respectively for searching, and only mRNAs that could be retrieved in all the above three databases were included in the subsequent sublineages. Further, miRNAs without targeting mRNAs were excluded and lncRNA-miRNA-mRNA relationship pairs were integrated. Finally, the ceRNA network was visualized by Cytoscape software.

### 2.9 Functional enrichment analysis

To reveal the molecular mechanism of the ceRNA network, we extracted the mRNAs from the network and conducted a functional enrichment analysis using the R package, clusterProfiler. Gene Ontology (GO) analysis was performed through the gseGO function in the clusterProfiler package. GO analysis includes three main categories, biological process (BP), cellular component (CC), and molecular function (MF). Kyoto Encyclopedia of Genes and Genomes (KEGG) pathway enrichment analyses were also conducted by gseKEGG function in clusterProfiler package (Yu et al., 2012). The adjusted (adj.) *p* < 0.05 was set as the cut-off criteria.

### 2.10 Evaluation of immune cell infiltration

We used the original CIBERSORT (Chen et al., 2018) gene signature file LM22, which defines 22 immune cell subtypes, to analyze datasets from human LUAD tissues. CIBERSORT *p* < 0.05 was included. To analyze the significant differential expression of different cell types of immune cells, we used the Wilcoxon rank-sum test method to observe the difference between the high- and low-risk group (http://cibersort.stanford.edu/). *p* < 0.05 was the cut-off standard. To further understand the relationship between risk score and these different types of immune cell infiltration, the Pearson correlation coefficient was used to find the correlation between risk score and these differentially expressed types of immune cells. The expression of immune checkpoint modulator (PD-L1) in the high- and low-risk groups was also investigated.

### 2.11 Immune checkpoint and tumor immune microenvironment (TIME) analyses

We obtained 43 immune checkpoint inhibitors (ICIs) from the published study (Supplementary Table S2), and investigated their expression levels between the high- and low-risk groups (Xie et al., 2022). The expression levels of the immune checkpoints between two risk groups were compared using Wilcoxon test. The immune and stromal scores were generated by the ESTIMATE algorithm for each LUAD sample in the TCGA-LUAD cohort. ESTIMATE algorithm was conducted to estimate the stromal score (StromalScore), immune score (ImmuneScore), ESTIMATE score (ESTIMATEScore), and tumor purity of LUAD samples based on expression data. The associations between StromalScore, ImmuneScore, ESTIMATEScores, tumor purity, and risk score were further analyzed using Spearman analysis respectively. Moreover, GSVA package was employed to explore the immune function difference between two risk groups (Hänzelmann et al., 2013). The immunologic signature gene sets (c7. all.v2022.1. Hs.symbols.gmt) were downloaded from MSigDB database (https://www.gsea-msigdb.org/gsea/msigdb/index.jsp), subsequently, limma package was applied to explore the differences in immune pathways with |T |>2 and adjust *p* < 0.05 as the cut-off values.

### 2.12 Tumor mutational load (TMB) profiles

We obtained mutation data from TCGA-LUAD and conducted a Wilcoxon rank-sum test to compare the TMB level of LUAD samples in the high- and low-risk groups. Furthermore, we analyzed the mutation type, the top 15 mutated genes, and the associations across the mutated genes in these groups using the maftools package (Mayakonda et al., 2018).

### 2.13 Patient and tissue preparation

Nine patients who underwent radical lung cancer surgery in Yunnan Cancer Hospital from September 2021 to November 2021 were included in this study. Inclusion criteria: 1. Patients did not receive preoperative antitumor therapy such as radiotherapy, chemotherapy, or targeted therapy; 2. Patients had complete clinical data and follow-up data; 3. Patients were pathologically diagnosed with primary non-small cell lung cancer; 4. Patients or family members signed an informed consent form and agreed to specimen collection, which was reported to the Ethics Committee of Yunnan Cancer Hospital for approval. Exclusion criteria: 1. Patients with primary non-small cell lung cancer combined with other tumors; 2. Patients with metastatic lung cancer; 3. Patients with combined chronic wasting diseases and infectious diseases.

The isolated specimens were collected within 10 min after surgical resection, and a portion of the specimens was placed in 4% paraformaldehyde solution immediately after obtaining for storage, while the remaining tissues were stored in a deep low temperature refrigerator at −80°C for real-time fluorescence quantitative PCR. The cancerous tissue and the normal lung tissue adjacent to cancer 3–5 cm from the edge of the cancer were confirmed by HE staining. In this study, patients were followed up by a regular return to the hospital for review or by telephone or WeChat, and the survival time of patients was 40 months starting from the first postoperative day to the patient’s death or the last follow-up visit. This study was approved by the Ethics Committee of Yunnan Cancer Hospital, and all cases participating in this study were informed and signed the informed consent form (approval number: KYLX2022176).

### 2.14 RNA isolation and quantitative real-time polymerase chain reaction (qRT-PCR)

All tumor tissue were lysed with TRIzol Reagent (Life Technologies-Invitrogen, Carlsbad, CA, United States), and total RNA was isolated following the manufacturer’s instructions. Then the concentration and purity of the RNA solution were quantified using a NanoDrop 2000FC-3100 nucleic acid protein quantifier (Thermo Fisher Scientific, Waltham, MA, United States Life Real). The extracted RNA was reverse-transcribed to cDNA using the SureScript-First-strand-cDNA-synthesis-kit (Genecopoeia, Guangzhou, China) prior to qRT-PCR. The qRT-PCR reaction consisted of 3 µL of reverse transcription product, 5 µL of 5×BlazeTaq qPCR Mix (Genecopoeia, Guangzhou, China), and 1 µL each of forward and reverse primer. PCR was performed in a BIO-RAD CFX96 Touch TM PCR detection system (Bio-Rad Laboratories, Inc., United States) under the following conditions: initial denaturation at 95°C for 1 min, followed by 40 cycles that each involved incubation at 95°C for 20 s, 55°C for 20 s, and 72°C for 30 s. The forward primer of LINC00460 was “TAA​GTG​CCC​GAA​TAA​AAG​G.” The reverse primer of LINC00460 was “GGA​TGG​CTC​AGG​AAA​AAC​A.” The forward primer of SYNPR-AS1 was “GCT​AAC​CAG​AGA​CAA​CCA​GT.” The reverse primer of SYNPR-AS1 was “TGT​GCC​TAT​CAA​TAA​AGA​GA.” The forward primer of RBPMS-AS1 was “ACA​CAA​AAA​CAC​ACG​CTC​C.” The reverse primer of RBPMS-AS1 was “GCC​AGT​TAA​GGC​AAT​CAA​T.” The forward primer of LINC00857 was “GAA​AAG​ACA​CCA​AAC​TCG​G.” The reverse primer of LINC00857 was “CTC​ATA​CAC​TCA​ACC​CAG​C.” The forward primer of GAPDH was “CCC​ATC​ACC​ATC​TTC​CAG​G.” The reverse primer of GAPDH was “CAT​CAC​GCC​ACA​GTT​TCC​C.” All primers were synthesized by Servicebio (Servicebio, Wuhan, China). The GAPDH gene served as an internal control, and the relative expression of 6 prognostic genes was determined using the 2^−ΔΔCt^ method (Livak and Schmittgen, 2001). The experiment was repeated in triplicate on independent occasions. Statistical differences of 4 prognostic lncRNAs between paracancer samples and LUAD samples were detected by paired t-tests, using GraphPad Prism V6 (GraphPad Software, La Jolla, CA, United States), and the level of statistical significance was tested and represented as * for *p* < 0.05; ** for *p* < 0.01.

### 2.15 Statistical analysis

All bioinformatics analyses in this study were performed in R software. Pearson correlation analysis was performed using the R package psych (Revelle and Revelle, 2015). The Cox regression analysis and survival analysis were performed in the R package survival. The ROC curves were plotted by the R package pROC (Robin et al., 2011). The most significant GO terms and KEGG pathways were visualized by the GOenrich package with a bubble/bar diagram (Yu, 2021). Unless otherwise stated, *p* < 0.05 was set as the threshold of significance.

## 3 Results

### 3.1 Identification of LUAD-related ARGs

**FIGURE 1:**
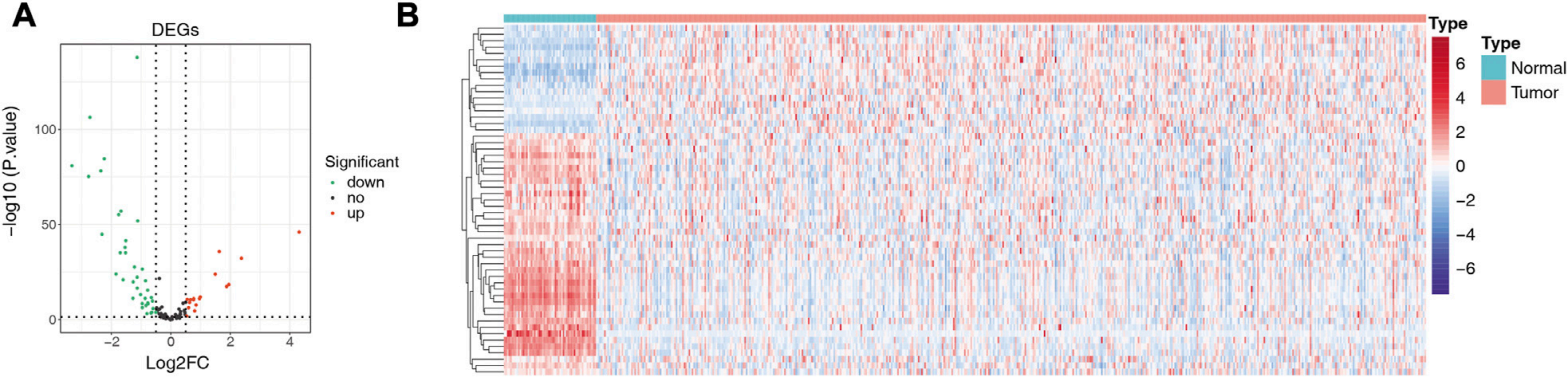
Detection of differentially expressed genes (DEGs). **(A)** Volcano plot showing DEGs between LUAD and adjacent normal tissues. **(B)** Heat map showing the expression pattern of all LUAD-related ARGs in normal and tumor groups; the *X*-axis indicates sample categories and *Y*-axis indicates DE-ARGs. Blue and red indicate down- and upregulation, respectively.

We matched the expression profiles of 104 ARGs in 58 normal and 491 LUAD samples from the TCGA database. Differential expression analysis was performed by R package limma. A total of 55 DE-ARGs were identified between the two groups (LUAD vs. normal; |log_2_ FC| > 0.5 and *p* < 0.05), in which 17 upregulated and 38 downregulated genes were found in LUAD relative to normal samples (Figure 1A; Supplementary Table S3). These DE-ARGs were considered LUAD-related ARGs. The heatmap demonstrated the expression pattern of all LUAD-related ARGs in normal and tumor groups (Figure 1B).

### 3.2 Identification of LUAD-related DE-lncRNAs

**FIGURE 2:**
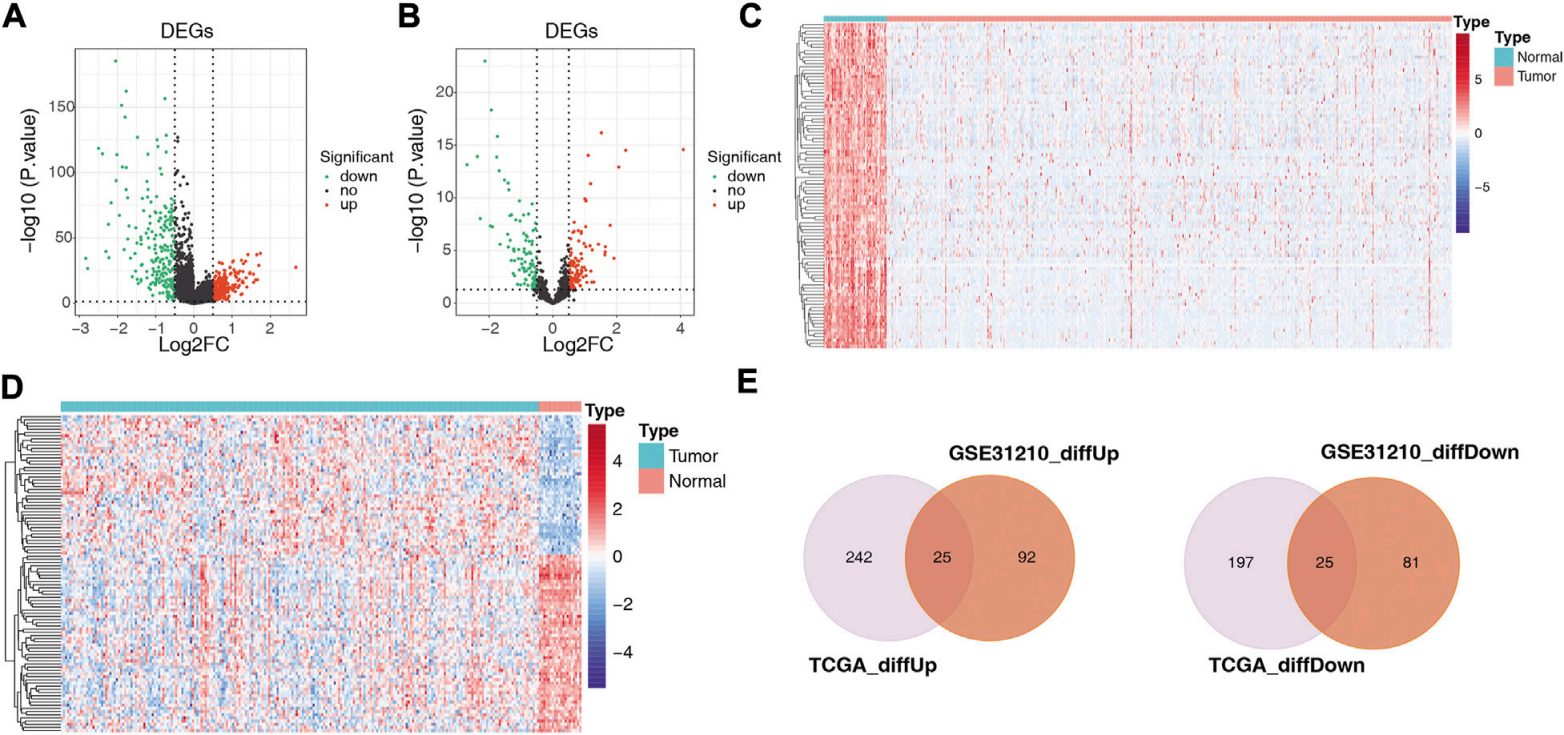
Volcano plots showing differentially expressed genes between LUAD and adjacent normal tissues in **(A)** 489 TCGA-DE-lncRNAs and **(B)** 223 GSE31210-DElncRNAs, respectively; heat maps showing **(C)** the top 100 TCGA-DE-lncRNAs and **(D)** top 100 GSE31210-DE-lncRNAs sorted according to *p*-value (ascending order), respectively GSE31210-DE-lncRNAs expression patterns in normal and tumor groups; **(E)** upper panel shows the intersection with DE-lncRNAs upregulated in the TGCA and GSE31210 datasets. The lower panel shows the intersection with the DE-lncRNAs downregulated in the TGCA and GSE31210 datasets.

A total of 489 TCGA-DE-lncRNAs were identified by R package limma based on lncRNA sequencing data from 549 samples (58 normal and 491 LUAD samples) in the TCGA database (LUAD vs. normal; |log_2_ FC| > 0.5 and *p* < 0.05; Supplementary Table S4). There were 267 genes significantly upregulated in the LUAD group and 222 genes significantly downregulated in the LUAD group compared with normal samples (Figure 2A). A similar analysis was conducted on the GES31210 dataset which had 20 normal and 226 LUAD samples). A total of 223 GSE31210-DE-lncRNAs were acquired, of which, 117 were upregulated and 106 were downregulated (Figure 2B; Supplementary Table S5). The heat map shows the expression patterns of the top 100 genes sorted according to *p*-value (ascending order) between the two groups in the TCGA dataset (Figure 2C) and the GES31210 dataset (Figure 2D), respectively. To obtain relatively accurate DE-lncRNAs, we performed overlap analysis on DE-lncRNAs from different databases (Figure 2E). Ultimately, we achieved 25 upregulated lncRNAs and 25 downregulated lncRNAs (relative to normal samples), and these 50 lncRNAs (Table 1) were defined as LUAD-related DE-lncRNAs.

**TABLE 1:** List of 50 LUAD-related DE-lncRNAs.

Gene Name	Change
SYNPR-AS1	UP
ITGB2-AS1	
SNHG3	
TMEM99	
LINC00857	
PITPNA-AS1	
C20orf197	
AFAP1-AS1	
LINC00511	
MIAT	
NKX2-1-AS1	
LBX2-AS1	
DPP10-AS1	
SBF2-AS1	
PCAT6	
PRRT3-AS1	
LOXL1-AS1	
UCA1	
RHPN1-AS1	
LINC00460	
LINC00342	
LINC01426	
CLDN10-AS1	
MNX1-AS1	
LINC00467	
LINC00092	DOWN
RBPMS-AS1	
PCAT19	
LINC00551	
LINC01082	
MAGI2-AS3	
MGC27382	
LHFPL3-AS2	
EP300-AS1	
LINC00968	
HHIP-AS1	
FENDRR	
FGF14-AS2	
LINC01352	
RGS5	
SENCR	
MBNL1-AS1	
TBX5-AS1	
BANCR	
GATA6-AS1	
LINC00702	
ADIRF-AS1	
SFTA1P	
NAV2-AS2	
LINC00472	

### 3.3 Characterization of ARGs-related DE-lncRNAs in LUAD

To obtain lncRNAs associated with ARGs, we analyzed the Pearson correlation of LUAD-related ARGs with LUAD-related DE-lncRNAs. A total of 36 lncRNAs were obtained according to |cor| > 0.4 and *p* < 0.001 (Supplementary Table S6), including ADIRF-AS1, EP300-AS1, FENDRR, FGF14-AS2, GATA6-AS1, HHIP-AS1, LHFPL3-AS2, LINC00092, LINC00460, LINC00467, LINC00472, LINC00511, LINC00551, LINC00702, LINC00857, LINC00968, LINC01082, LINC01352, LINC01426, MAGI2-AS3, MBNL1-AS1, MGC27382, MNX1-AS1, NAV2-AS2, PCAT19, PCAT6, PITPNA-AS1, RBPMS-AS1, RGS5, RHPN1-AS1, SBF2-AS1, SENCR, SFTA1P, SNHG3, SYNPR-AS1, and TBX5-AS1. These lncRNAs were considered as angiogenesis-related DE-lncRNAs for further analysis.

### 3.4 Construction of angiogenesis-related lncRNAs signature

**FIGURE 3:**
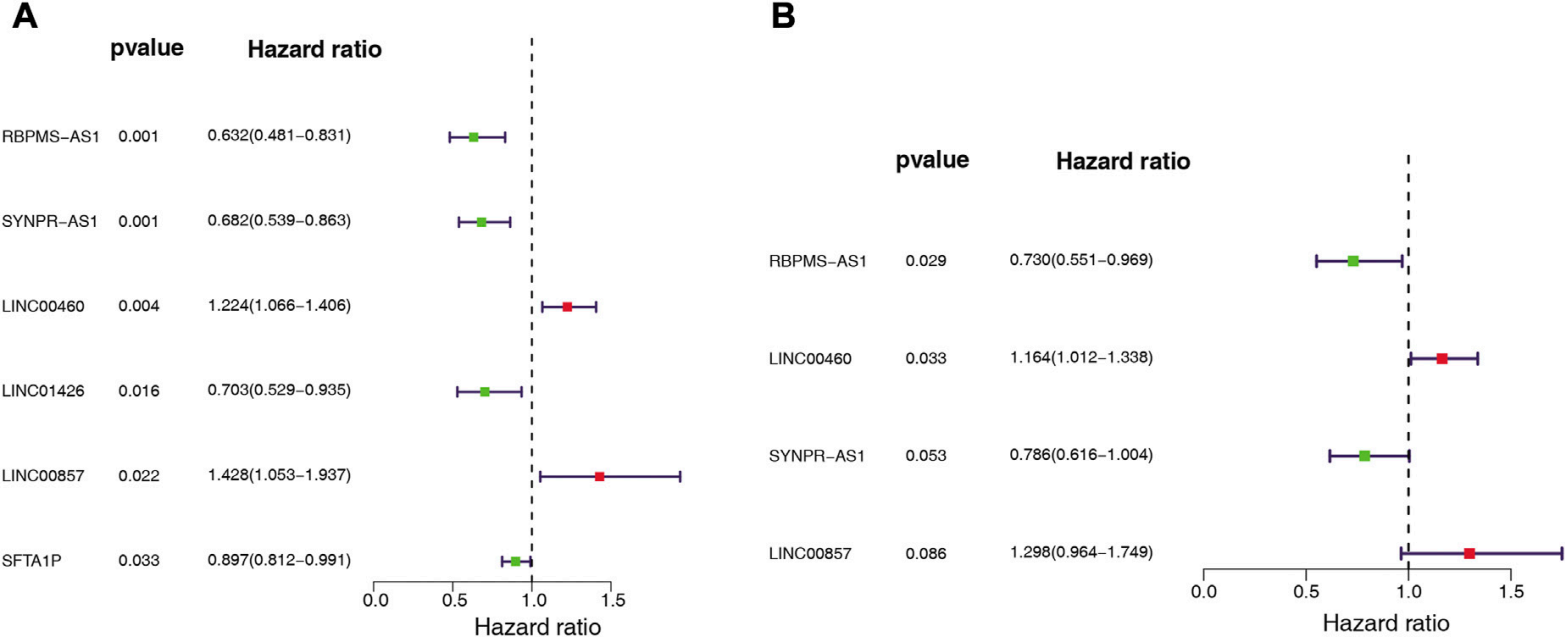
Whether ARG-associated DE-lncRNAs is a risk factor for prognosis in LUAD patients was analyzed by **(A)** univariate and **(B)** multivariate Cox regression in the TCGA cohort.

We assessed the prognostic value of the identified ARGs-related DE-lncRNAs in LUAD patients from the TCGA-training set (*n* = 343). Univariate Cox regression analysis was employed to investigate whether ARGs-related DE-lncRNAs were risk factors for prognosis in LUAD patients. Out of 36 lncRNAs, six were significantly associated with the prognosis of LUAD patients based on the cut-off value (Figure 3A). Among them, with HR > 1, LINC00857 (HR = 1.428, 95% CI: 1.053–1.937, *p* = 0.022) and LINC00460 (HR = 1.224, 95% CI: 1.066–1.406, *p* = 0.004) might be oncogenic factors for LUAD; while with HR < 1, RBPMS-AS1 (HR = 0.632, 95% CI: 0.481–0.831, *p* = 0.001), SYNPR-AS1 (HR = 0.682, 95% CI: 0.539–0.863, *p* = 0.001), LINC01426 (HR = 0.703, 95% CI: 0.529–0.935, *p* = 0.016), and SFTA1P (HR = 0.897, 95% CI: 0.812–0.991, *p* = 0.033) were then expected to be potential protective factors for LUAD. Further, these prognosis-related lncRNAs of LUAD have integrated into a stepwise regression multivariate Cox regression analysis. Ultimately, LINC00857, LINC00460, SYNPR-AS1, and RBPMS-AS1 were suggested as the optimal variables for the construction of prognostic signature (Figure 3B; Supplementary Table S7). Consequently, we developed an angiogenesis-related lncRNAs signature based on the above four lncRNAs.

### 3.5 Evaluation and validation of the 4-lncRNAs-based risk score

**FIGURE 4:**
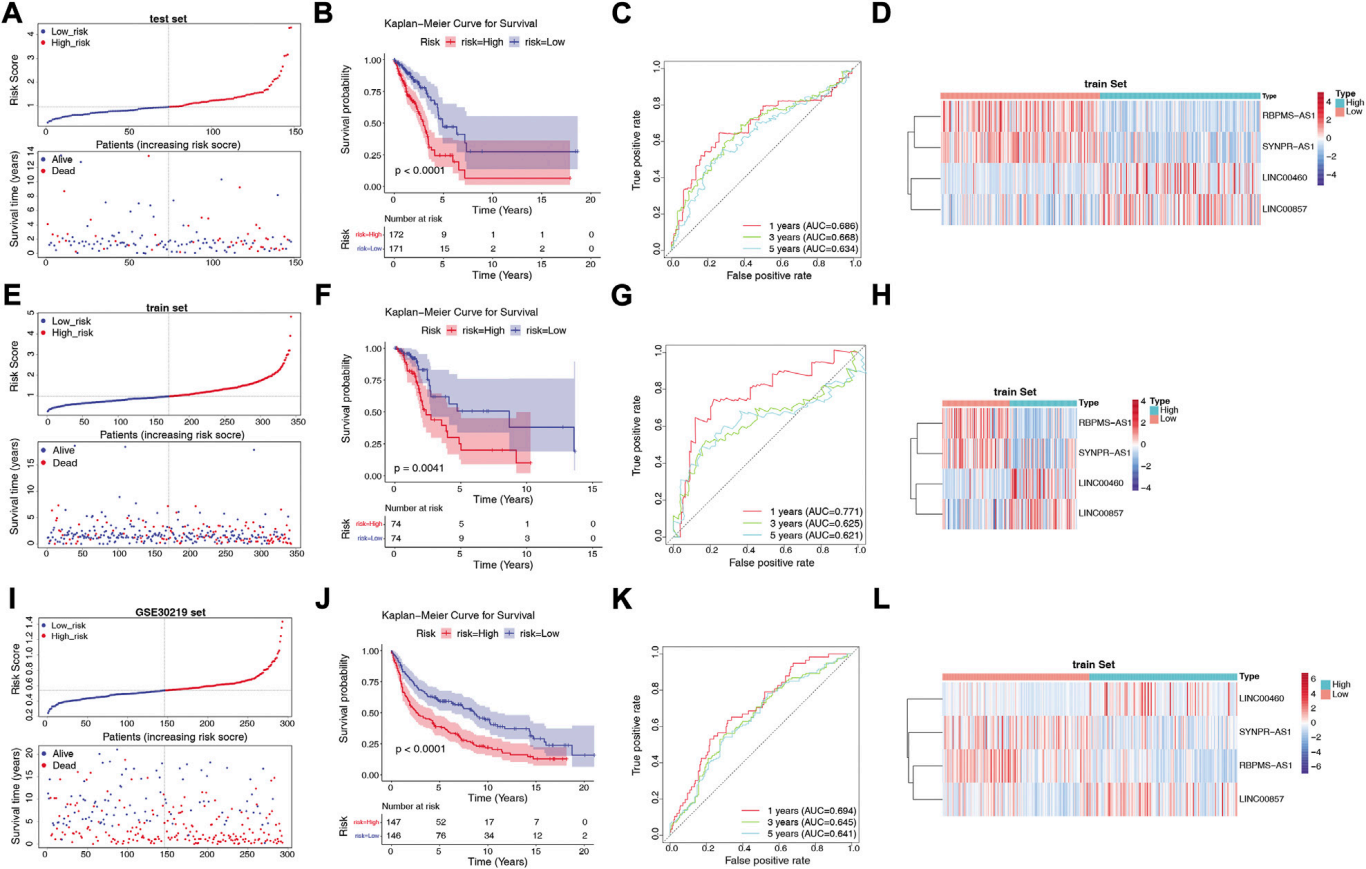
Construction and validation of the risk model. **(A)** Distribution of risk scores and distribution of OS in the LUAD sample in the TCGA training set. **(B)** Kaplan-Meier survival analysis of OS between the high-risk and low-risk groups in the TCGA training set. **(C)** AUC in ROC analysis of 1-year, 3-year and 5-year survival time risk profiles in the TCGA training set. **(D)** Expression patterns of RBPMS-AS1, SYNPR-AS1, LINC00460 and LINC00857 in the high-risk and low-risk groups in the TCGA training set. **(E)** Distribution of risk scores and distribution of OS in the LUAD samples in the TCGA test set. **(F)** Kaplan-Meier survival analysis of OS between the high-risk and low-risk groups in the TCGA test set. **(G)** AUC in the ROC analysis of risk characteristics for 1-year, 3-year, and 5-year survival times in the TCGA test set. **(H)** Expression patterns of RBPMS-AS1, SYNPR-AS1, LINC00460 and LINC00857 in the high-risk and low-risk groups in the TCGA test set. **(I)** Distribution of risk scores and distribution of OS in the LUAD sample in the GSE30219 dataset. **(J)** Kaplan-Meier survival analysis of OS between the high-risk and low-risk groups in the GSE30219 dataset. **(K)** AUC in ROC analysis of 1-year, 3-year, and 5-year survival time risk profiles in the GSE30219 dataset. **(L)** Expression patterns of RBPMS-AS1, SYNPR-AS1, LINC00460, and LINC00857 in the high-risk and low-risk groups in the GSE30219 dataset.

We assessed the predictive validity of the prognostic signature in the TCGA-training set (*n* = 343). Based on the formula described in Materials and Methods, we calculated risk scores for patients in the TCGA-training set. According to the median value of the risk score (median value = 0.9661), 172 LUAD samples were classified in the high-risk group and 171 samples were in the low-risk group (Supplementary Table S8). From Figure 4A, we found that the number of deaths in LUAD patients increased with a progressive increase in the patient’s risk score. The K-M survival curve revealed that the risk score significantly differentiated the clinical outcomes of patients, with a high-risk score implying a poorer likelihood of survival (*p* < 0.0001; Figure 4B). The AUC of the ROC curve for risk score in predicting patients’ OS at 1, 3, and 5 years was 0.686, 0.668, and 0.634, respectively (Figure 4C), suggesting that the risk score has a certain degree of prognostic predictive validity. Moreover, the expression patterns of prognostic lncRNAs in the two risk groups were demonstrated in Figure 4D, RBPMS-AS1 and SYNPR-AS1 were highly expressed in the low-risk group; while LINC00460 and LINC00857 were highly expressed in the high-risk group.

Next, we validated the validity of the signature in the TCGA-test set (*n* = 148) and the GSE30219 dataset (*n* = 293). Similarly, the LUAD samples in both the TCGA-test set (Supplementary Table S9) and the GSE30219 dataset (Supplementary Table S10) were classified into high- and low-risk groups based on the corresponding median values. The prognostic signature based on the four lncRNAs in both validation sets was consistent with their performance in the TCGA-training set. The risk curves and patient survival status in the TCGA-test set and GSE30219 dataset were shown in Figures 4E, I, respectively, with the majority of the patients who died clustered in the high-risk group. In the GSE30219 dataset, the survival time of patients with low-risk scores was significantly longer. The K-M curves further demonstrated that patients in the low-risk group had better OS compared to the high-risk group (Figures 4F, J). In the TCGA-test set, the AUCs at 1, 3, and 5 years were 0.711, 0.625, and 0.621, respectively (Figure 4G). In the GSE30219 dataset, the AUC of the risk score in predicting patients’ OS at 1 year was 0.694, 0.645 at 3 years, and 0.641 at 5 years (Figure 4K). The expression pattern of prognostic lncRNAs in the high- and low-risk groups of the validation set (Figures 4H, L) was consistent with their expression in the TCGA-training set.

### 3.6 Risk score is an independent prognostic factor for LUAD

**FIGURE 5:**
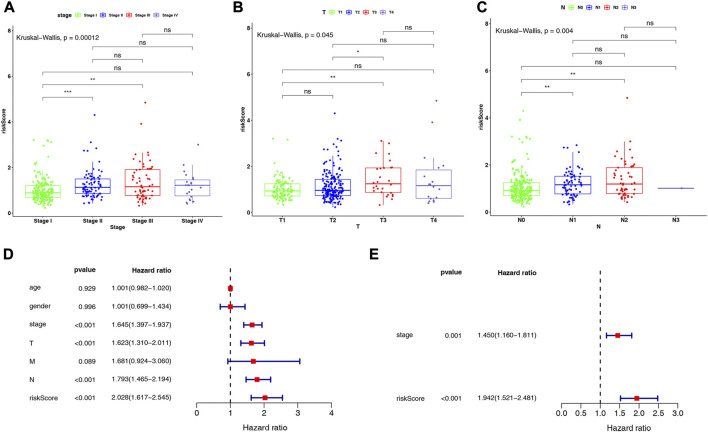
Differences in risk scores in **(A)** stage subgroups, **(B)** T-stage subgroups, and **(C)** N-stage subgroups. Results of **(D)** univariate and **(E)** multivariate Cox regression analyses on clinical characteristics of LUAD in the TCGA-LUAD dataset.

We investigated the distribution of risk scores in LUAD clinical subgroups to determine their association with disease characteristics. The results showed that in the stage subgroup, overall, the risk score increased with stage progression and was significantly higher in patients in stage II and III groups compared with stage I (Figure 5A). In the T-stage subgroup, the highest risk score was observed in the T3 group, which was significantly higher than in the T1 and T2 groups (Figure 5B). In the N-stage subgroup, the risk score was significantly higher in the N1 and N2 groups compared to the N0 group, but no significant difference was observed between the N1 and N2 groups (Figure 5C). However, no direct association was found between risk score and age, gender, or M-stage (Supplementary Figure S1).

Further, we conducted Cox regression analysis to determine whether the risk score could independently impact the prognosis of patients with LUAD, after considering clinical characteristics such as age, gender, stage, and TNM stage, based on the entire TCGA-LUAD dataset. Univariate Cox regression analysis indicated that stage, T-stage, N-stage, and risk score were significantly associated with patients with LUAD (all *p* < 0.001; Figure 5D). Subsequently, multivariate Cox regression analyses were performed to explore the independent prognostic effects of the above 4 variables. As shown in Figure 5E, stage (*p* = 0.001) and risk score (*p* < 0.001) were identified as independent prognostic factors for LUAD.

### 3.7 Construction and validation of a nomogram based on 4 prognostic lncRNAs

**FIGURE 6:**
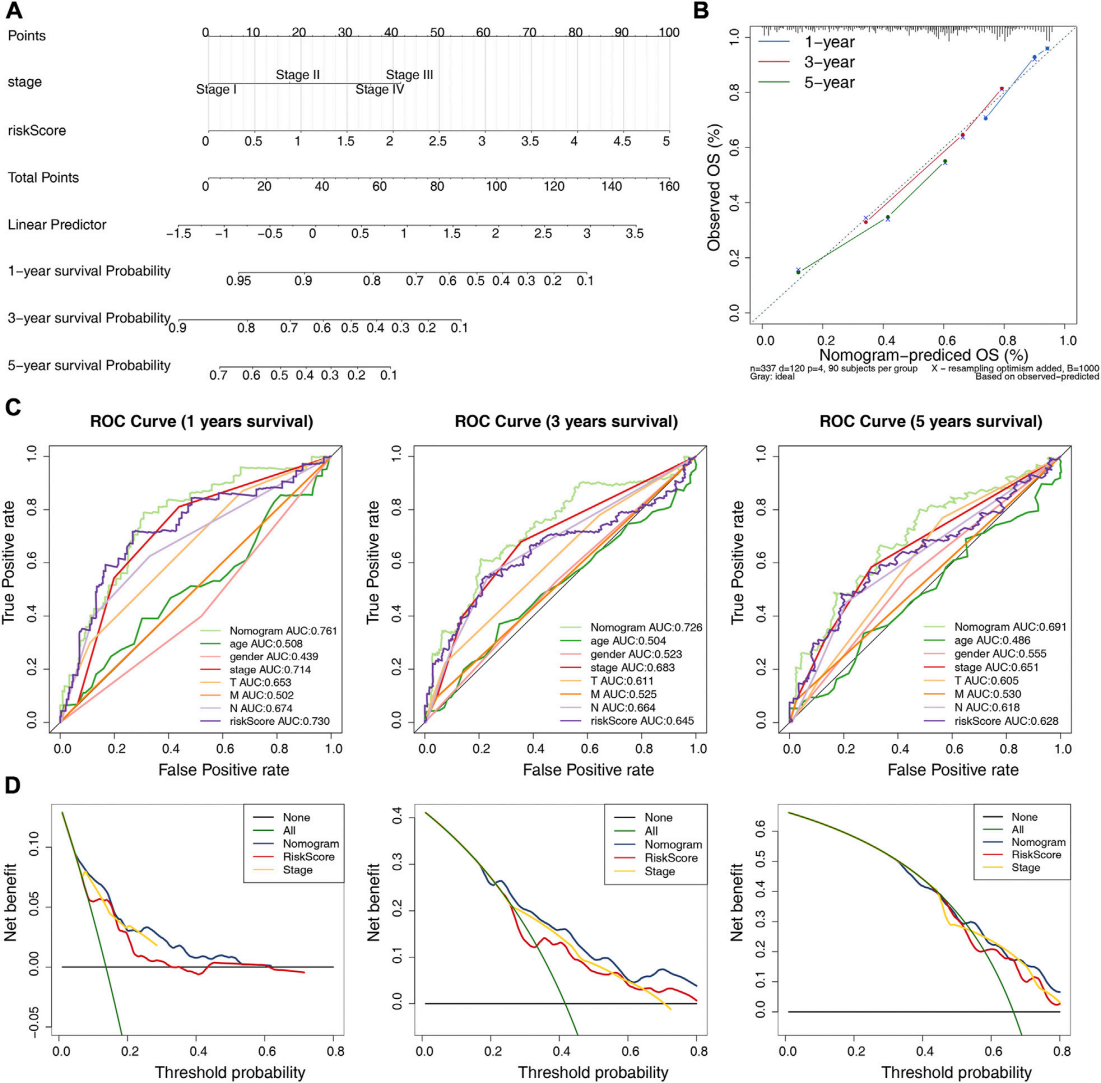
Establishment and validation of a nomogram. **(A)** A nomogram based on the riskscore and independent prognostic factors. **(B)** Calibration curve for the nomogram. **(C)** ROC curves of the nomogram and clinical characteristics (N and stage) at 1-, 3-, and 5-year. **(D)** DCA curve of the nomogram at 1-, 3-, and 5-year.

The independent prognostic factors (stage and risk score) were utilized to construct a nomogram (Figure 6A). The c-index of the nomogram was 0.729, indicating the nomogram with favorable discrimination. The calibration plot indicated a high consistency between the nomogram in predicting and observing survival probability (Figure 6B). The ROC curves indicated that the nomogram was superior to the clinical characteristics (age, gender, stage, and pathologic TMN stage) and risk score in terms of prognosis with the AUC values of the nomogram at 1-, 3- and 5-year were 0.761, 0.726, and 0.691, respectively (Figure 6C). The DCA curves demonstrated that the prognostic performance of the nomogram was mostly satisfactory at 3-year survival (Figure 6D). Overall, these results suggested that the nomogram based on four ARGs-related prognostic lncRNAs was an efficient method to predict the prognosis of LUAD patients.

### 3.8 Analysis of the ceRNA network based on ARGs-related prognostic lncRNAs

**FIGURE 7:**
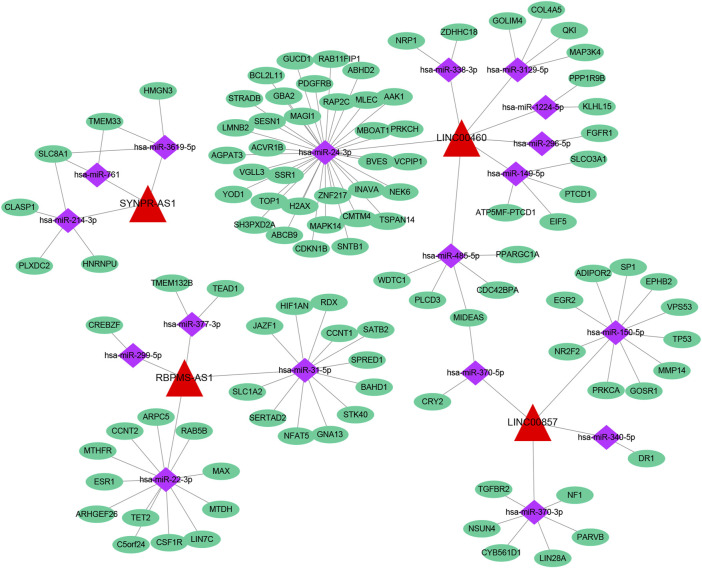
A lncRNA-miRNA-mRNA ceRNA network containing 125 nodes and 125 edges.

**FIGURE 8:**
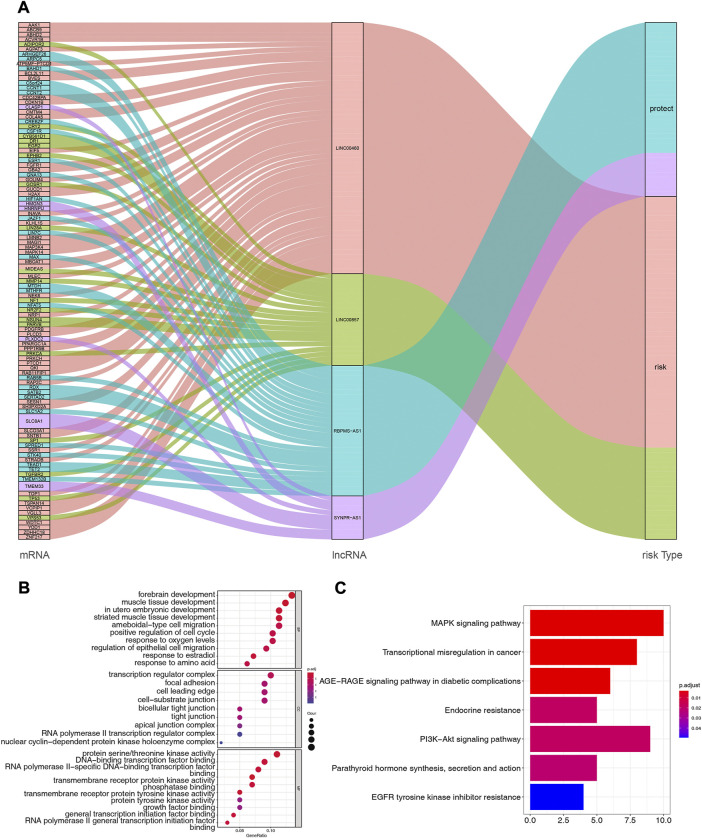
**(A)** Correspondence of 103 mRNAs regulated by ceRNA network with 4 prognostic lncRNAs. **(B)** Functional enrichment analysis of 103 mRNAs in the ceRNA network, the graph represents GO-BP, GO-CC and GO-MF terms from top to bottom, respectively. **(C)** Pathway enrichment analysis of mRNAs in the ceRNA network (7 enriched pathways are shown).

Based on multiple public databases, we eventually visualized a lncRNA-miRNA-mRNA ceRNA network containing 125 nodes (4 prognostic lncRNAs, 18 miRNAs, and 103 mRNAs) and 125 edges by Cytoscape software (Figure 7). LINC00460 could competitively bind 8 miRNAs (hsa-miR-1224-5p, hsa-miR-149-5p, hsa-miR-24-3p, hsa-miR-296-5p, hsa-miR-3129-5p, hsa-miR-338-3p, and hsa-miR-485-5p) to regulate the expression of 52 mRNAs. LINC00857 regulated the expression of 19 mRNAs through four sponge miRNAs (hsa-miR-150-5p, hsa-miR-340-5p, hsa-miR-370-3p, and hsa-miR-370-5p); RBPMS-AS1 regulated the expression of 19 mRNAs through four miRNAs (hsa-miR-22- 3p, hsa-miR-299-5p, hsa-miR-31-5p, and hsa-miR-377-3p) to form a ceRNA machinery with 27 mRNAs; SYNPR-AS1 modulated 9 mRNAs by competitively binding hsa-miR-214-3p, hsa-miR-3619-5p, and hsa-miR-761 mRNAs expression. Overall, RBPMS-AS1 and SYNPR-AS1 have relatively independent ceRNA mechanisms, but LINC00857 and LINC00460 could simultaneously regulate MIDEAS through the competitive binding to hsa-miR-5p and hsa-miR-485-5p, respectively. The specific predicted ceRNA mechanisms could be reviewed in Supplementary Table S11. Moreover, the correspondence of 103 mRNAs regulated in the ceRNA network with 4 prognostic lncRNAs was demonstrated by the Sankey diagram (Figure 8A; Supplementary Table S12). The 70 mRNAs regulated by LINC00460 (targeting 52 mRNAs) and LINC00857 (targeting 19 mRNAs) may be risk factors for LUAD, whereas the 33 mRNAs regulated by RBPMS-AS1 (targeting 27 mRNAs) and SYNPR-AS1 (targeting 6 mRNAs) may be protective factors for LUAD.

Subsequently, we performed functional enrichment analysis for 103 mRNAs in the ceRNA network. The top 10 GO-BP and -MF terms and all GO-CC terms were displayed in Figure 8B. In the GO-BP category, these genes were significantly associated with “forebrain development,” “muscle tissue development,” and “*in utero* embryonic development.” Meanwhile, biological processes related to the cell cycle, such as “positive regulation of cell cycle,” “organelle fission, “regulation of mitotic nuclear division,” and “nuclear division,” were also highly enriched. Unexpectedly, terms related to angiogenesis biological process such as “vasculogenesis,” “coronary vasculature morphogenesis,” “regulation of blood vessel endothelial cell migration,” “branching involved in blood vessel morphogenesis,” “cranial nerve development,” and “positive regulation of angiogenesis” appeared strongly enclosed. Furthermore, these genes were involved in biological processes related to respiratory development, such as “lung development,” “respiratory tube development,” and “respiratory system development,” as well as oxygen response processes, such as “response to oxygen levels,” “response to hypoxia,” and “response to decreased oxygen levels.” Additionally, “positive regulation of macrophage migration,” “myeloid leukocyte differentiation,” “cell-substrate adhesion,” “macrophage migration,” and “positive regulation of macrophage chemotaxis” were also closely linked to these genes. Furthermore, these genes may perform the molecular functions of “protein serine/threonine kinase activity,” “DNA-binding transcription factor binding,” and “RNA polymerase II-specific DNA-binding transcription factor binding” in cellular components such as “transcription regulator complex,” “focal adhesion,” “cell leading edge.” Detailed GO analysis results were available in Supplementary Table S13. KEGG analysis enriched a total of 7 pathways (Figure 8C; Supplementary Table S14), “MAPK signaling pathway,” “Transcriptional misregulation in cancer,” and “AGE-RAGE signaling pathway in diabetic complications” were the most enriched significant (all adj. *p* = 0.003068) pathways. Among them, the “MAPK signaling pathway” was the pathway in which the most mRNAs in the ceRNA network were involved (count = 10).

### 3.9 Immune landscape analysis of risk score-based LUAD

**FIGURE 9:**
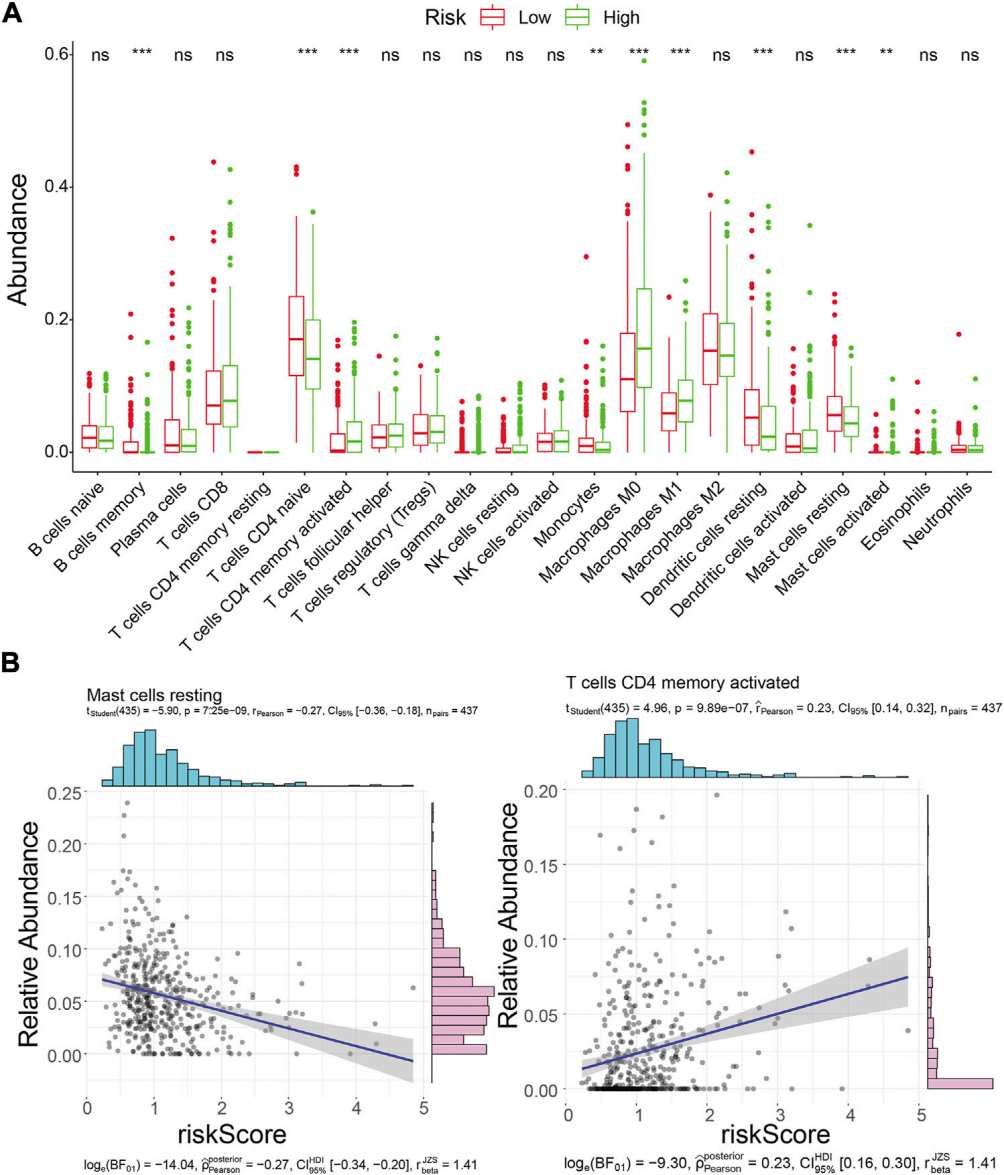
Immune landscape analysis. **(A)** Immune landscape of LUAD patients in the high- and low-risk groups. Differences in immune cell infiltration and immune-related pathways between risk groups. ns: not significant; *p < 0.05; * * *p < 0.01; * * *p < 0.001; * * * * *p < 0.0001.**(B)** Correlation analysis of risk scores with activated memory CD4 T Cells and mast cell resting abundance in the TCGA training cohort.

Inspired by the functional enrichment analysis showing that mRNAs targeted by prognostic lncRNAs were associated with immune cells, we assessed the immune landscape of LUAD patients in the high- and low-risk groups using the CIBERSORT algorithm (Figure 9A). The results showed that B Cells memory, T Cells CD4 memory resting, Monocytes, Dendritic cells resting, and Mast cells resting were significantly associated with the low-risk group (all *p* < 0.01). On the other hand, T Cells CD4 memory activated, Macrophages M0, Macrophages M1, and Mast cells activated were more abundant in the high-risk group (all *p* < 0.01). Further correlation analysis (Figure 9B; Supplementary Table S15) showed that the risk score was only weakly correlated with T Cells CD4 memory activated (cor = 0.23, *p* = 9.89E-07) and Mast cells resting (cor = −0.27, *p* = 7.25E-09). These results suggested that the risk score seemed not to be directly involved in altering the tumor microenvironment but may instead carry out its regulatory function on tumor immune cells through ceRNA mechanisms, which warrants further corroboration.

### 3.10 ICIs and TIME

**FIGURE 10:**
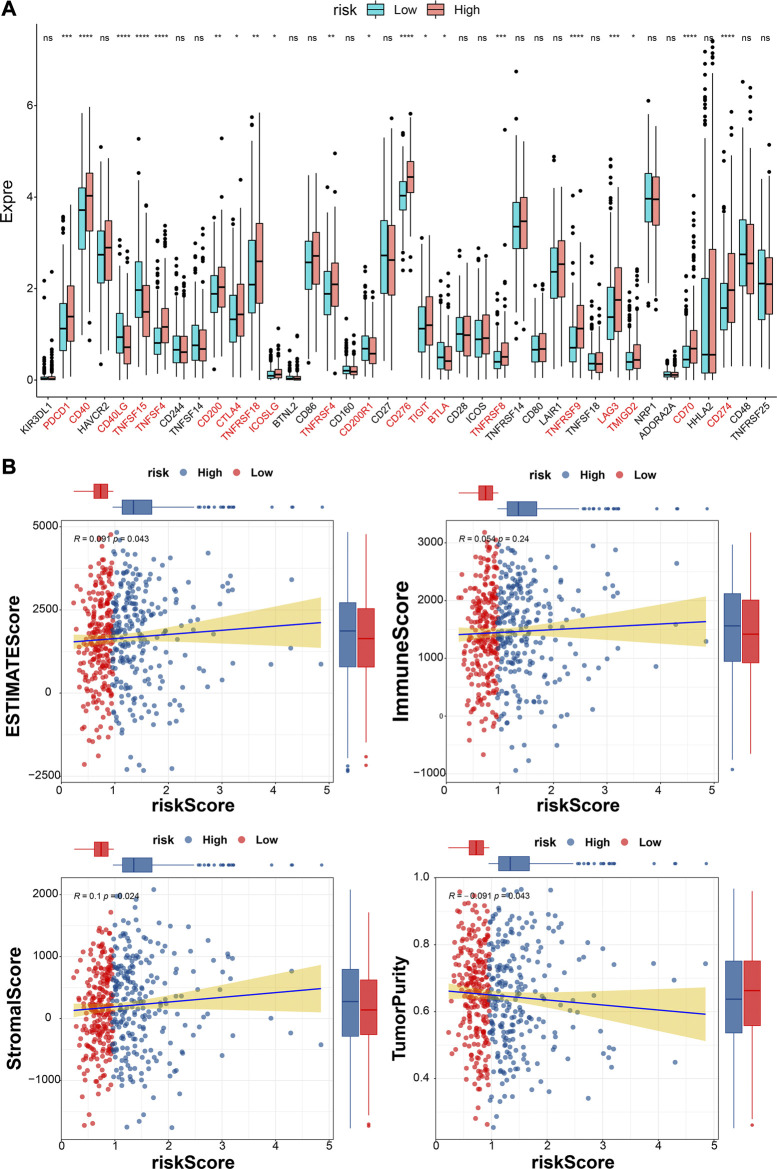
Analyses of immune checkpoint and tumor immune microenvironment. **(A)**The expressions of ICIs between high- and low-risk groups (Wilcox test). * represents *p* < 0.05, ** represents *p* < 0.01, *** represents *p* < 0.001, **** represents *p* < 0.0001, and ns indcates no significance. **(B)** The correlations between estimate score, immune score, stromal score, tumor purity, and risk score.

The differences in the expression of 43 ICIs between high- and low-risk groups were analyzed, and the results indicated that the expressions of 20 ICIs were significant differences between high- and low-risk groups (*p* < 0.05), including PDCD1, CD40, CD40LG, TNFSF15, and TNFSF4 et al. (Figure 10A). Furthermore, the ESTIMATEScore (R = 0.091, *p* = 0.043) and StromalScore (R = 0.1, *p* = 0.024) were significantly positively correlated to risk scores, while the tumor purity was negatively correlated to risk score (R = − 0.091, *p* = 0.043) (Figure 10B). The immune function difference analysis showed 2937 pathways were significantly different between high- and low-risk groups (Supplementary Table S16).

### 3.11 Differences in PD-L1 expression and TMB between two risk subgroups

**FIGURE 11:**
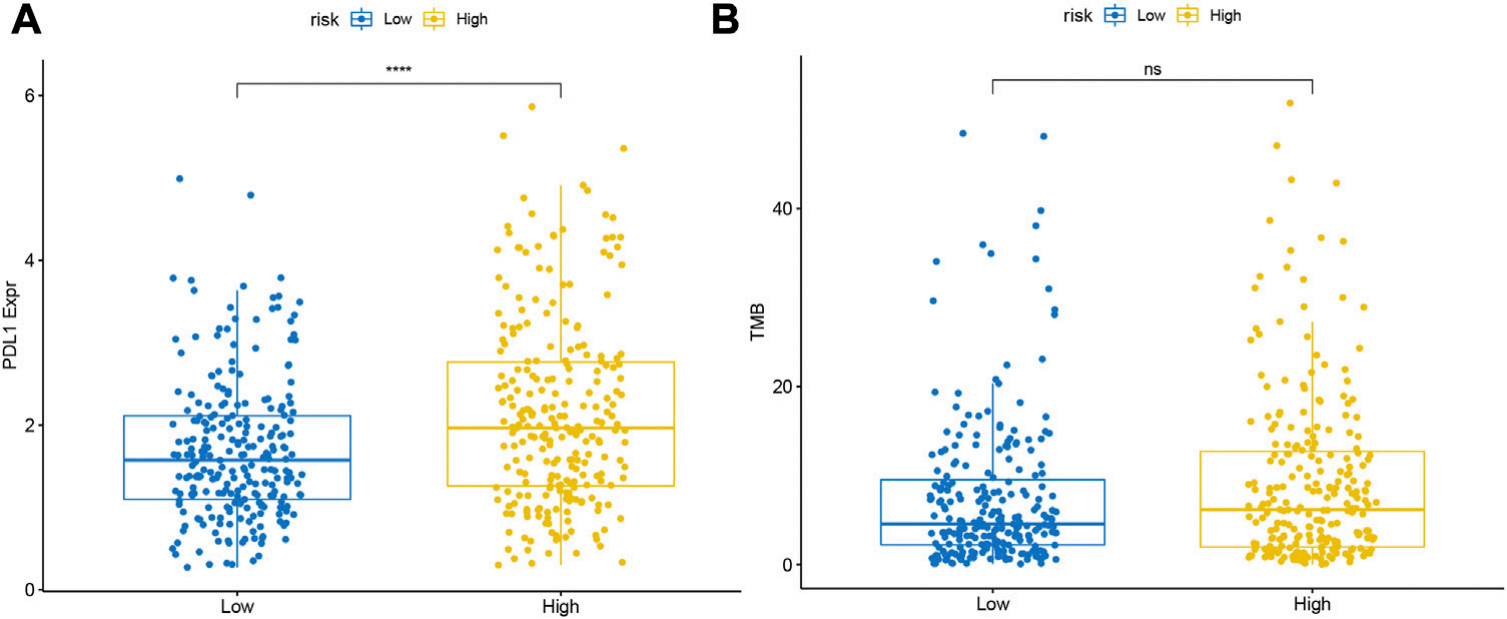
Differences in the expression levels of **(A)** PDL1 and **(B)** TMB between the high- and low-risk groups.

In the TCGA database, the level of PD-L1 expression was significantly higher in the high-risk group than in the low-risk group (Figure 11A). Meanwhile, we found that samples in the high-risk group of TCGA-LUAD had higher TMB, although it was not statistically significant (Figure 11B).

### 3.12 Comparison of mutation characteristics between high- and low-risk groups

**FIGURE 12:**
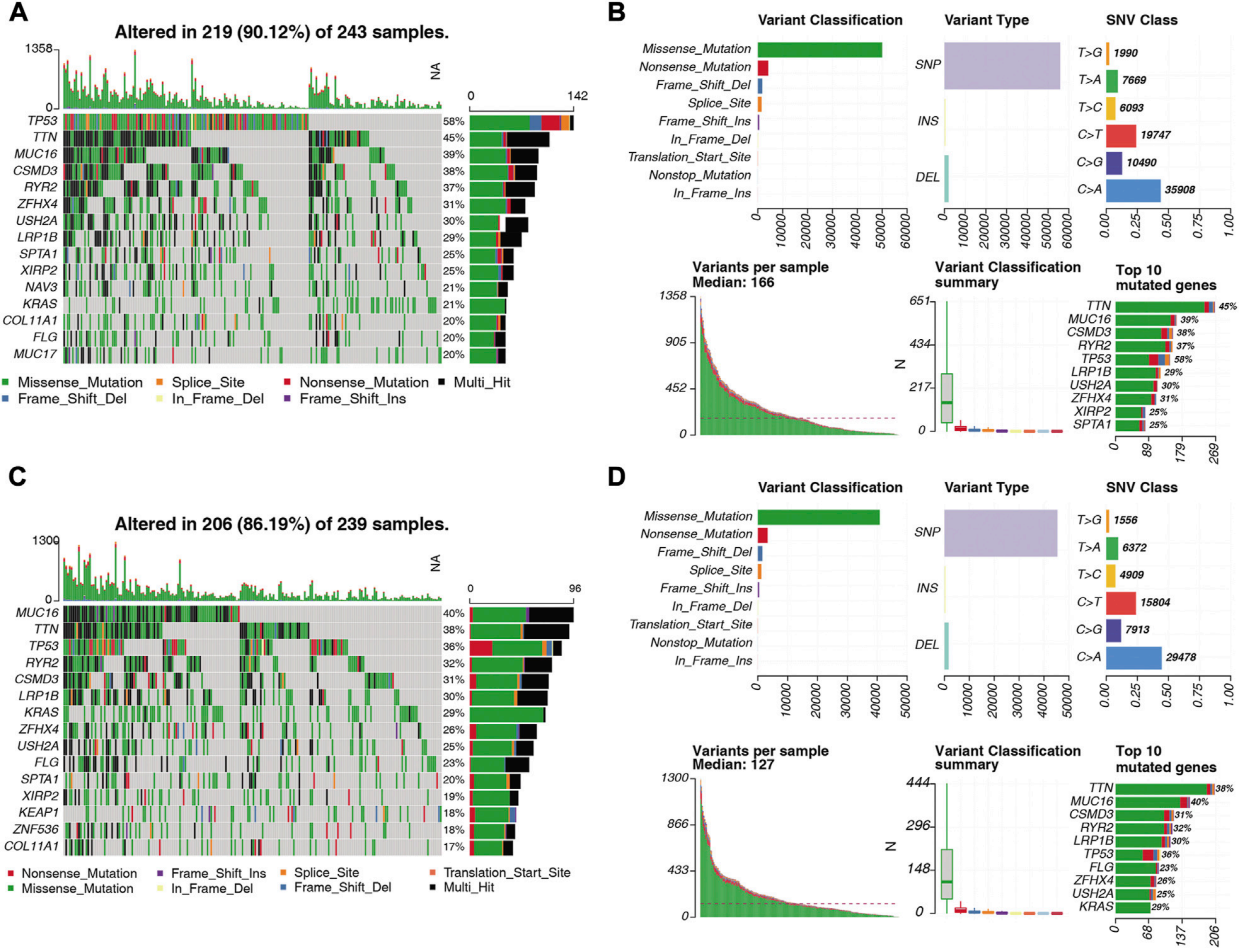
Distribution of somatically mutated genes and SNV types in LUAD samples in the **(A)** high-risk and **(C)** low-risk groups. **(B)** Variant classification of somatic cells in LUAD samples in the high-risk and **(D)** low-risk groups. Classification, variant type, SNV class, variants per sample, variant classification summary statistics.

We visualized the somatic mutation details of each LUAD sample in the high- and low-risk groups by waterfall charts and observed that the top 15 mutated genes in the two subgroups were slightly different, but mainly TP53, TTN, and MUC16 were in the top three (Figures 12A, C). We found that TP53 (58%) had the highest mutations in the high-risk population, while MUC16 (58%) had the highest mutations in the low-risk population. For Variant Classification, missense mutations were common in other mutations. For different Variant Types, SNP was responsible for most of the variants, and single nucleotide variants (SNV) mostly appeared on C > A (Figures 12B, D).

### 3.13 Validation of the expression of prognostic lncRNAs in clinical LUAD tissues

**FIGURE 13:**
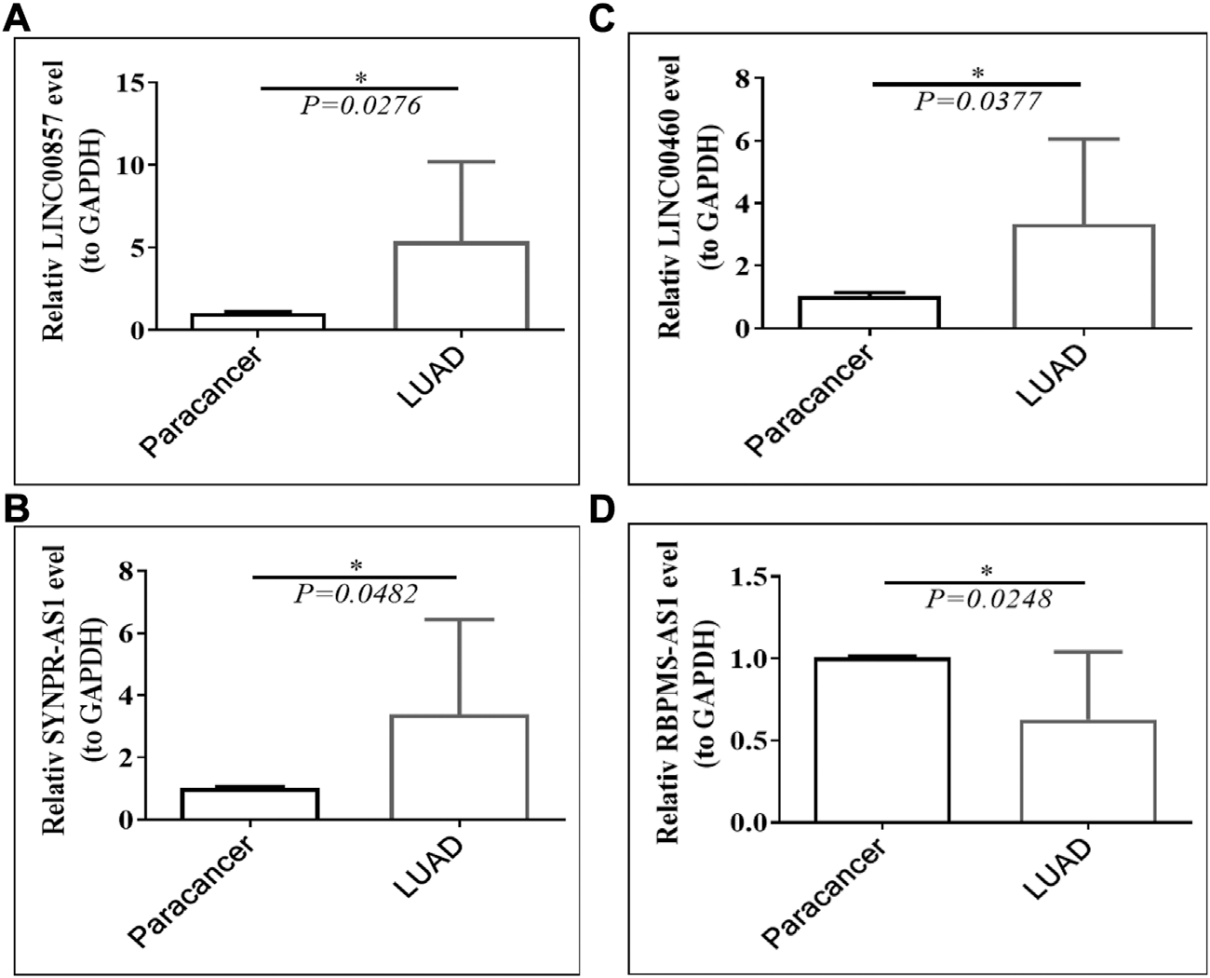
Expression of **(A)** LINC00857, **(B)** SYNPR-AS1, **(C)** LINC00460 and **(D)** RBPMS-AS1 in LUAD tissues and paraneoplastic tissues.

Public database expression analysis showed that LINC00857, SYNPR-AS1, and LINC00460 were upregulated in TCGA-LUAD (Supplementary Table S4) and GSE30219-LUAD (Supplementary Table S5), and RBPMS-AS1 was downregulated. We obtained nine pairs of tumor tissue samples and paracancerous samples of LUAD patients from Yunnan Cancer Hospital, and the patient information is shown in Table 2. The expression levels of four prognostic lncRNAs by qRT-PCR, the original data from the PCR instrument were shown in Supplementary Material. The melting curves of four prognostic lncRNAs and GAPDH were exhibited in Supplementary Figure S2. As illustrated in Figure 13, LINC00857, SYNPR-AS1, and LINC00460 were significantly more expressed in the LUAD group than in the paracancerous tissues (all *p* < 0.05), while RBPMS-AS1 was more highly expressed in the paracancerous tissues relative to the LUAD tissues (*p* = 0.0248). These results were consistent with the expression trends in the two public databases.

**TABLE 2:** | Clinicopathologic characteristics of 9 LUAD patients.

Parameter		Total (*n*=9)
Gender	Male	5 (55.6%)
	Female	4 (44.4%)
Age (years)	<60	6 (66.7%)
	≥60	3 (33.3%)
Tumor size (cm)	<3	4 (44.4%)
	≥3	5 (55.6%)
Tumor location	Left	2 (22.2%)
	Right	7 (77.8%)
Histological grade	Middle or low	4 (44.4%)
	High	5 (55.6%)
Smoking history	Yes	4 (44.4%)
	No	5 (55.6%)
Staging	I	6 (66.7%)
	II	2 (22.2%)
	III	1 (11.1%)

## 4 Discussion

Lung adenocarcinoma is a common and deadly disease, with the number of new cases and deaths remaining high and even increasing each year (Bade and Dela Cruz, 2020). In the current study, the prognostic characteristics of six angiogenesis-related lncRNAs, LINC00857, LINC01462, SFTA1P, RBPMS-AS1, SYNPR-AS1 and LINC00460, were constructed, and the validity of the models was assessed and found to correlate with clinical characteristics using K-M and ROC curves. A prognostic lncRNA-miRNA-mRNA -ceRNA network was identified. The pathway analysis found that “MAPK signaling pathway” is the pathway involving the most mRNAs in the ceRNA network. Immune cell infiltration and mutation characterization revealed that risk scoring may complete its regulatory role on tumor immune cells through ceRNA mechanism, and SNP is the cause of most of the mutations, and SNVs are mostly found on C>A. The expression of LINC00857, RBPMS-AS1, SYNPR-AS1 and LINC00460 was validated in clinical cases. Subsequently, we constructed a Cox risk model with good predictive performance based on these four human angiogenesis-associated lncRNAs, which may be an independent prognostic predictor of LUAD.

Angiogenesis is the process of germination from existing blood vessels to form new ones, which plays a crucial role in the carcinogenesis of lung adenocarcinoma (Wang Y. et al., 2019). Numerous studies have shown the prognostic value of ARGs in cancer (Zheng et al., 2021; Cai et al., 2022; Tao et al., 2022), where Sun et al. found that POSTN may ultimately affect the prognosis of patients with LUAD by altering the immune microenvironment (Sun et al., 2022), and in our study, 55 ARGs associated with LUAD were obtained from 549 samples in the TCGA database. In addition, we also identified LUAD-related DE-lncRNAs and obtained 50 LUAD-related DE-lncRNAs. lncRNAs are a class of non-coding RNAs longer than 200 nucleotides, and lncRNAs may have regulatory roles in a variety of biological functions (Zhu et al., 2013; Jarroux et al., 2017). Some studies suggest that lncRNAs possess important value in the prognosis and immunotherapy of LUAD (Xu et al., 2021). Therefore, we performed a Pearson correlation analysis of LUAD-associated ARG with LUAD-associated DE-lncRNAs and obtained 36 ARG-associated lncRNAs. It has been shown that ARG-associated lncRNAs are closely associated with the survival of STAD patients (Han et al., 2021) and it has also been shown that ARG-associated lncRNAs can predict the prognosis of hepatocellular carcinoma patients (Lei et al., 2021), however, the value of ARG-associated lncRNAs in LUAD has yet to be investigated.

To further investigate the prognostic value of lncRNAs for LUAD, we employed univariate Cox regression analysis to explore whether ARG-related DE-lncRNAs were risk factors for the prognosis of LUAD patients. We found that six DE-lncRNAs significantly associated with the prognosis of LUAD patients, and ultimately screened out LINC00857, LINC00460, SYNPR-AS1 and RBPMS-AS1. It has been shown that LncRNA LINC00857 can regulate lung adenocarcinoma progression by targeting miR-1179/SPAG5 axis (Wang et al., 2020), LINC00460 -Hsa-Mir-338-FAM111/ZWINT pathway can be used as a prognostic biomarker for lung adenocarcinoma (Li et al., 2022), SYNPR-AS1 is significantly associated with overall survival of lung adenocarcinoma patients (Wu et al., 2020) and the prognostic potential of RBPMS-AS1 in lung adenocarcinoma (Wang L. et al., 2019), which is consistent with our findings. To assess the validity of prognostic features, we calculated patient risk scores in the TCGA training and prediction sets. Previous studies have indicated that lower tumor microenvironment-related risk scores may indicate better response and OS outcomes of immunotherapy in LUAD patients (Wu et al., 2021) and immune signature-based risk scores indicate that LUAD patients with low risk scores have significantly higher immune phenotype scores (Yi et al., 2021). In our study, we found that a predictive function of 4-lncRNAs-based risk scores was indicative of the prognosis of LUAD patients. In addition, we found that LINC00460 was highly expressed in the high-risk group, and similar findings were discovered in head and neck squamous cell carcinoma by Du et al. (Gong and Ning, 2020). Furthermore, we explored independent prognostic factors for LUAD, and univariate Cox regression analysis showed that stage, T-stage, N-stage, and risk score were significantly associated with patients with LUAD. Further multivariate Cox regression analysis identified stage and risk score as independent prognostic factors for LUAD. While a study based on alternative splicing (AS) genes found that LUAD risk characteristics were associated with gender and T, N and TNM staging (Zhao et al., 2021), another study based on autophagy-related genes in LUAD showed that risk score was strongly associated with T-stage, tumor stage and prognosis (Zhou et al., 2021).

In addition, we performed a ceRNA network analysis of ARG-related prognosis-based lncRNAs by Cytoscape software and found that RBPMS-AS1 and SYNPR-AS1 have relatively independent ceRNA mechanisms. However LINC00857 and LINC00460 can regulate MIDEAS simultaneously through competitive binding to hsa-miR-5p and hsa-miR-485-5p, respectively. Wu et al. conducted a similar study and constructed a LUAD-specific lncRNA-miRNA-mRNA ceRNA regulatory network containing 157 nodes and 378 edges. They showed by GO and KEGG pathways that the LUAD-specific ceRNA network is associated with tumor-related molecular functions and pathways (Wu et al., 2020). In our study, we found by KEGG analysis that the MAPK signaling pathway involves the most mRNAs in the ceRNA network.

The relevance of mRNAs targeted by lncRNAs in enrichment analysis to immune cells aroused our curiosity, prompting an investigation into the immune landscape of LUAD patients in high and low risk groups. We discovered that CD4 memory resting T Cells and resting dendritic cells were significantly associated with the low risk group, while in a related study on colorectal cancer, the low risk group had a higher percentage of CD4 memory resting T Cells, activated dendritic cells, and resting dendritic cell infiltration, as compared to the high-risk group (Li et al., 2020). However, a study on ovarian cancer yielded results that were opposite to ours. In their study, a higher proportion of CD4 memory resting T Cells was found in samples from patients with high-risk scores, with a large number of activated memory CD4 T Cells and M1 macrophages found in samples from the low-risk group (Su et al., 2021). This discrepancy may be due to the fact that their risk scores were established based on immune-related genes (IRGs) and transcription factors (TFs). To further assess the relationship between the risk score and immune prognosis, we examined the PD-L1 expression levels between the two risk subgroups and found that PD-L1 expression levels were significantly higher in the high-risk group than in the low-risk group, consistent with the fact that Luo et al. also showed significant differences in PD-L1 expression between the low- and high-risk groups in patients with LUAD (Luo et al., 2020). In addition, we compared the mutation characteristics of the high-risk and low-risk groups and found that TP53 mutations were more common in the high-risk population, which was also identified in one study that found a higher frequency of TP53 mutations in the C1 subtype of LUAD (Zheng et al., 2020). Finally, we validated the expression of prognostic lncRNAs in clinical LUAD tissues and found expression trends consistent with two public databases, with one study showing that LINC00857 expression was significantly upregulated in LUAD paraneoplastic tissues compared to normal lung tissue (Wang et al., 2020), which is consistent with our results.

In summary, we established and validated lncRNA prognostic models associated with angiogenesis in lung adenocarcinoma and determined the prognostic value of LINC00857, RBPMS-AS1, SYNPR-AS1 and LINC00460 in LUAD. We also discovered that the low-risk group was associated with a positive prognosis and that resting immune cells were more abundant in the low-risk group, though the expression of immune checkpoint molecules was lower. Moreover, validation in the clinic demonstrated that LINC00857, SYNPR-AS1 and LINC00460 were significantly overexpressed in tumor tissues and RBPMS-AS1 was highly expressed in paraneoplastic tissues. However, additional clinical cohorts and experiments are necessary to confirm the credibility of this study. Research on lung adenocarcinoma has a long way to go, and the present study strives to provide new insights for accurate treatment and prognostic assessment of lung cancer.

## Data Availability

The original contributions presented in the study are included in the article/Supplementary Material, further inquiries can be directed to the corresponding author.

## Supplementary material

SUPPLEMENTARY FIGURE S1 Correlation of risk scores with **(A)** age, **(B)** gender, and **(C)** M stage.

SUPPLEMENTARY FIGURE S2 The melting curves of four prognostic lncRNAs and GAPDH.
