# Supplementary figures and images for: Construction and validation of an angiogenesis-related lncRNA prognostic model in lung adenocarcinoma
Source: Front Genet. 2023 Mar 14;14:1083593. doi: 10.3389/fgene.2023.1083593 (PMC10043447; doi:10.3389/fgene.2023.1083593)

risk Low High

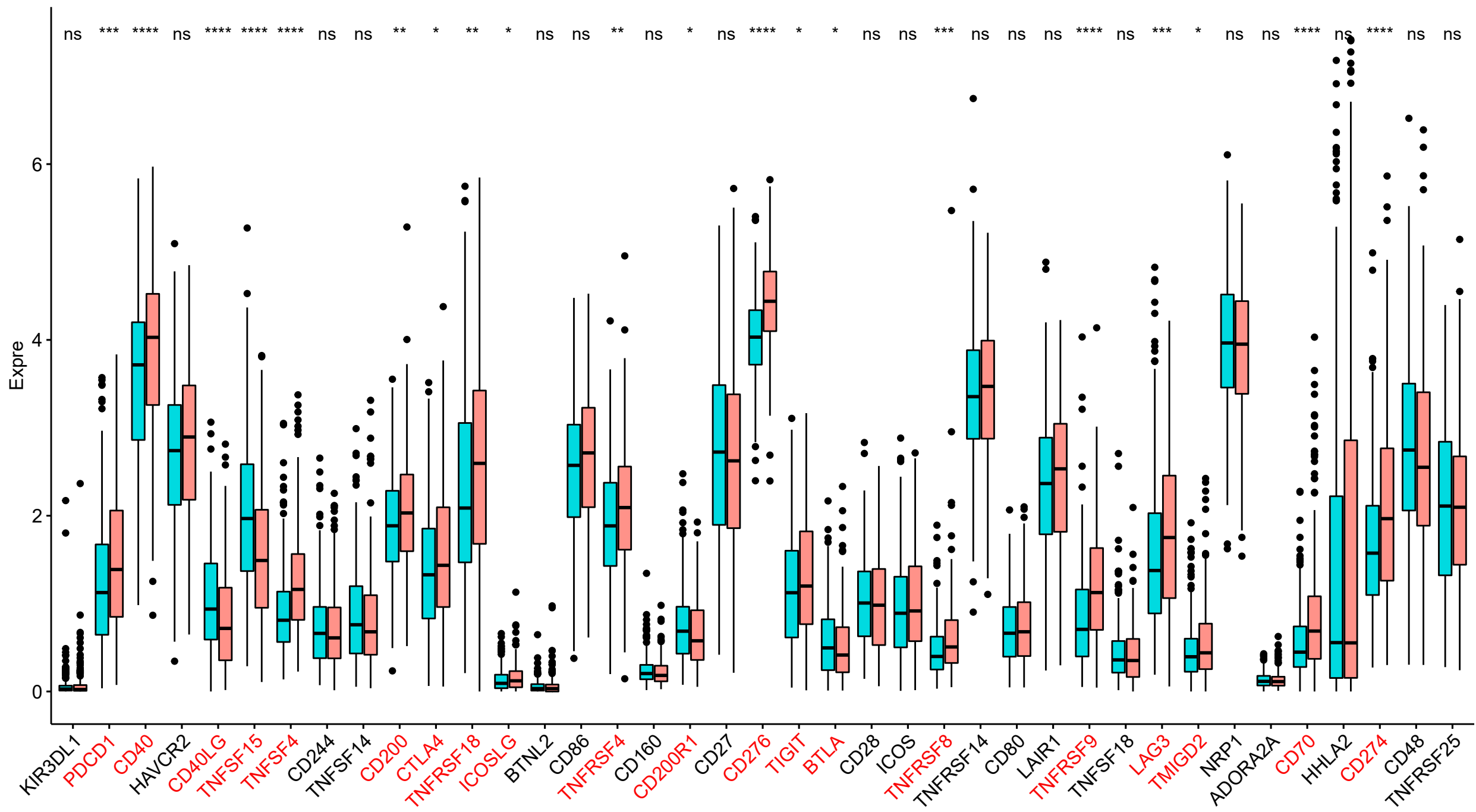

Supplement: Supplementary file 1 [file DataSheet2.PDF]

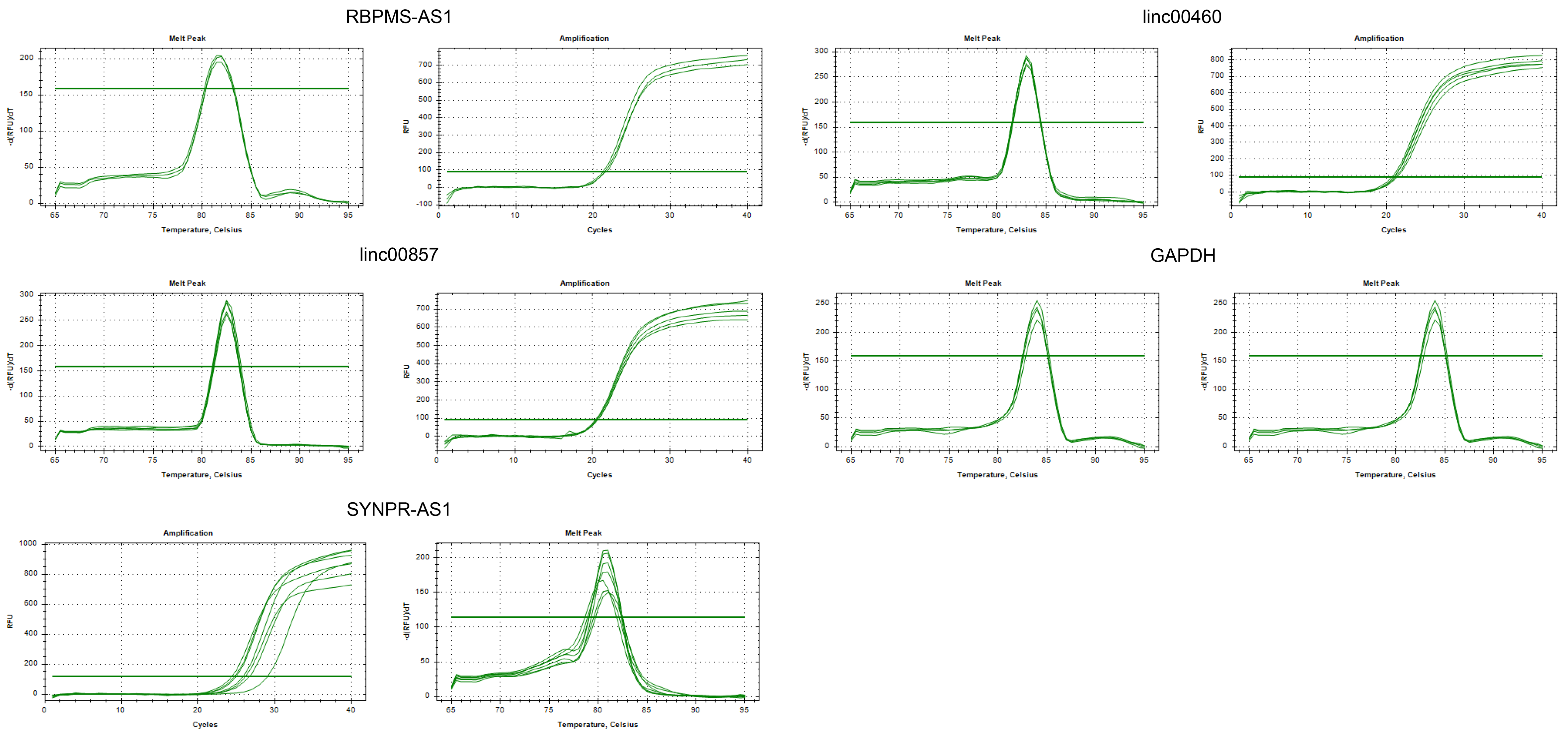

Supplement: Supplementary file 6 [file Image2.TIF]

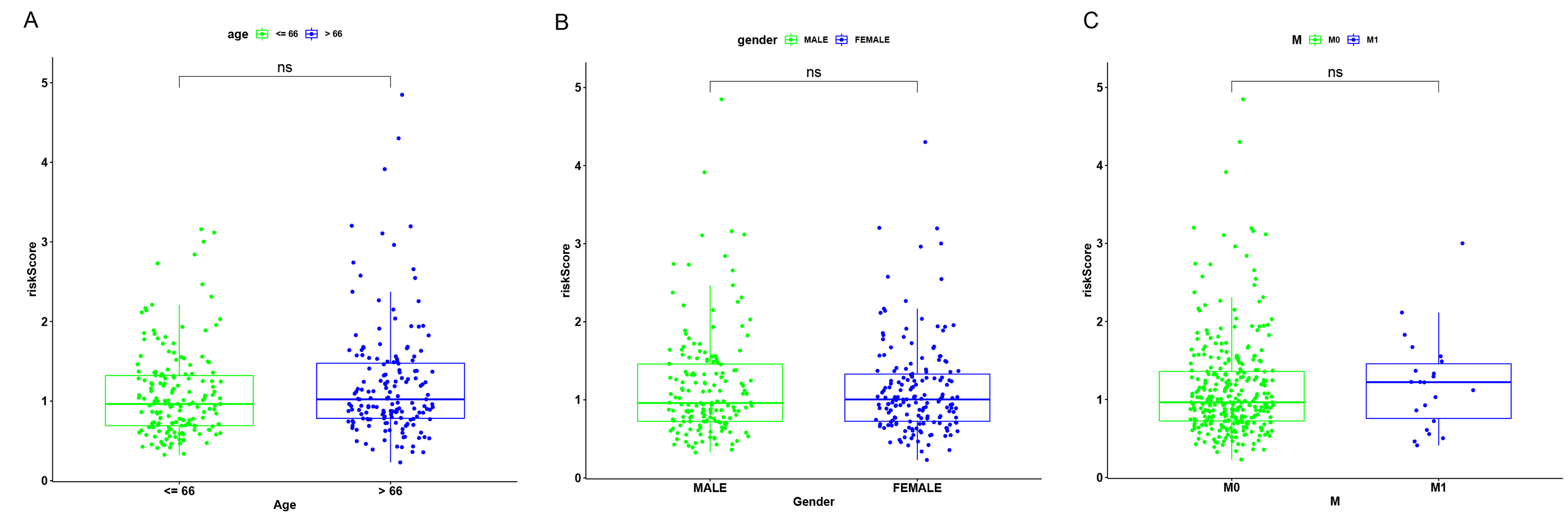

Supplement: Supplementary file 8 [file Image1.TIF]

RBPM5-AS1

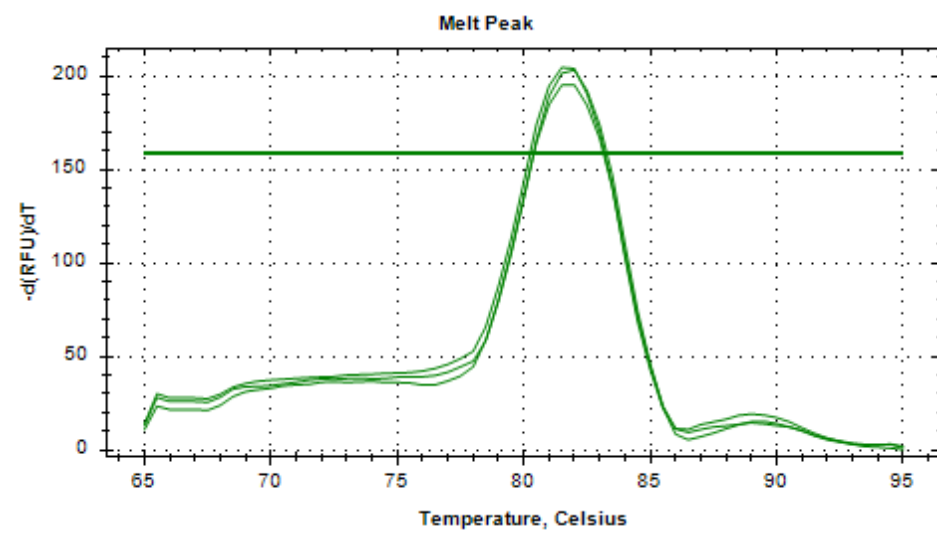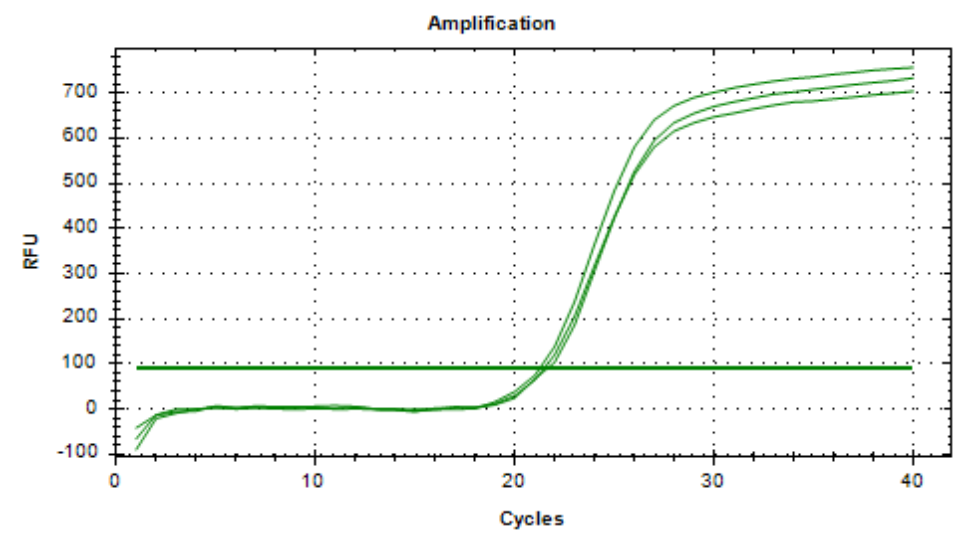

linc00460

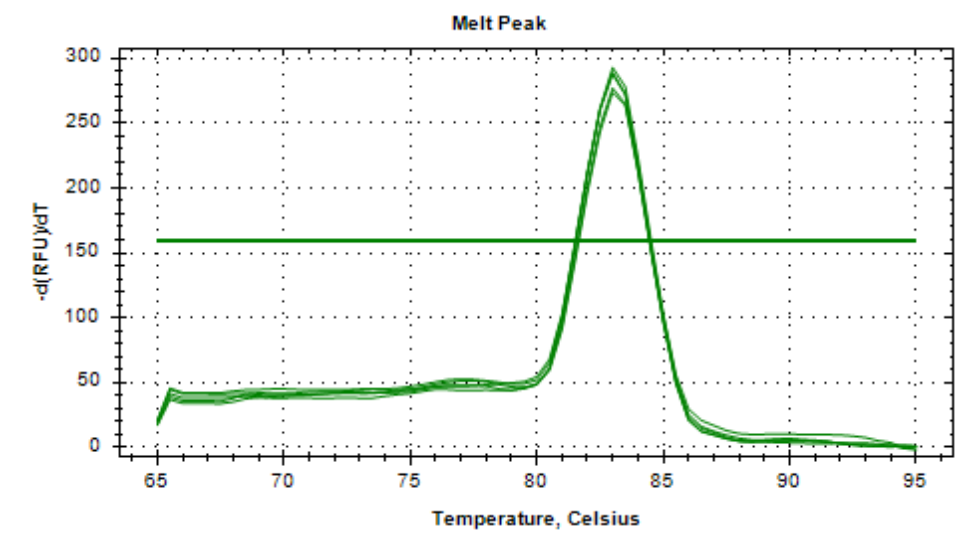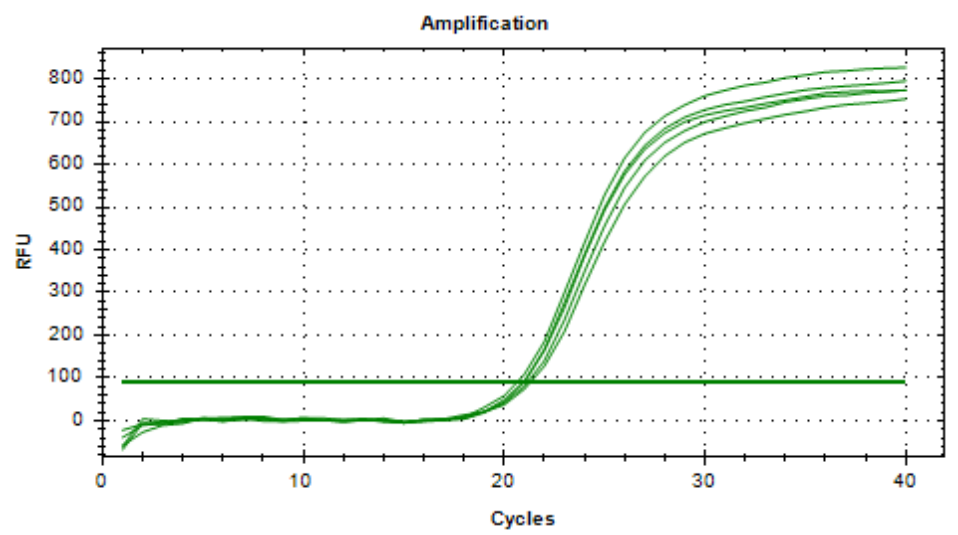

linc00857

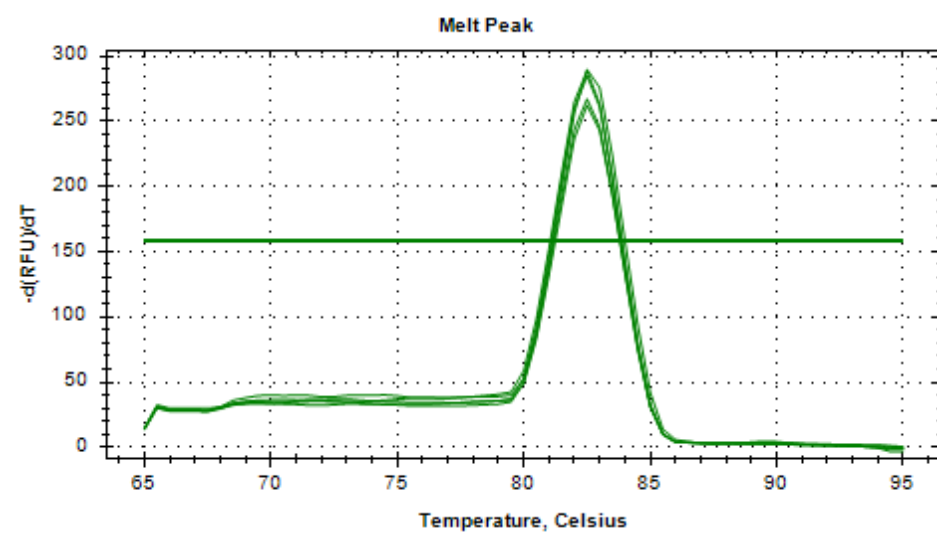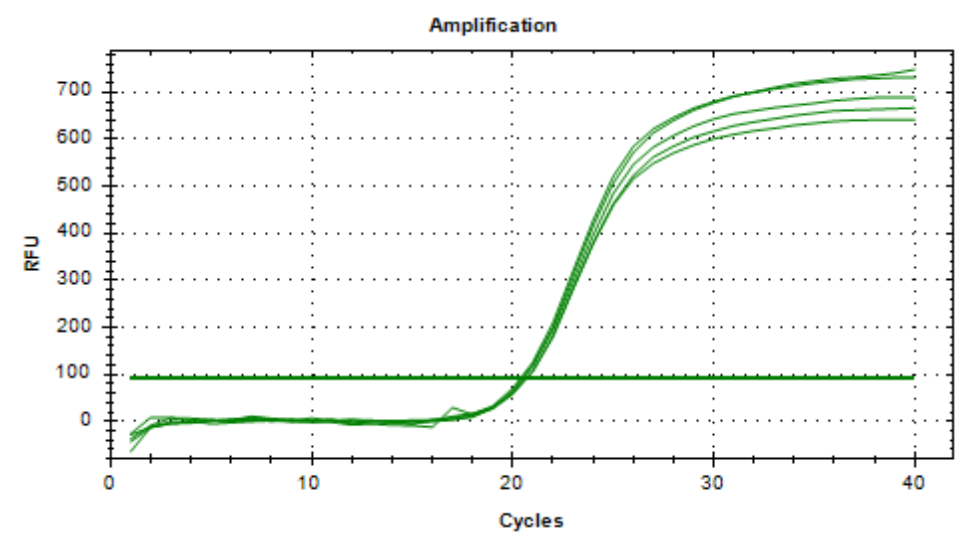

GAPDH

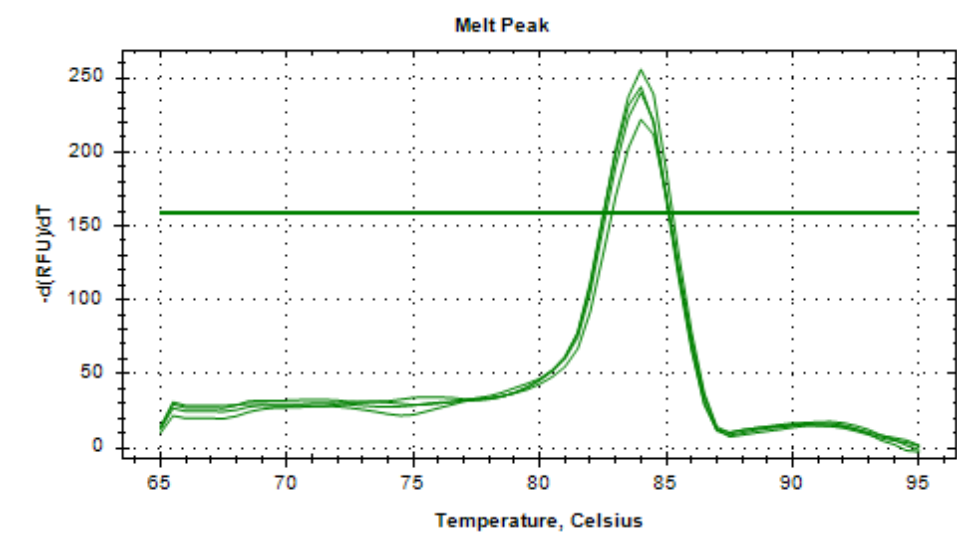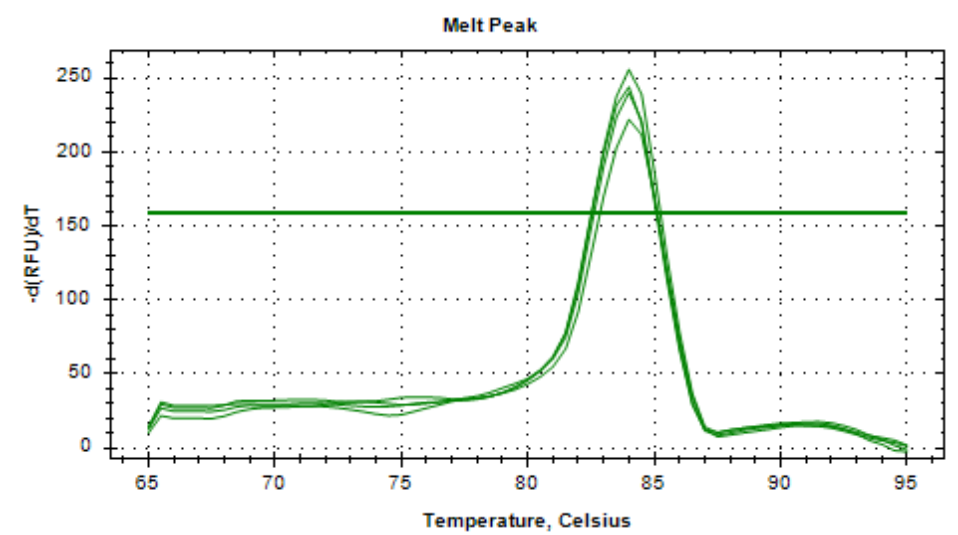

SYNPR-AS1

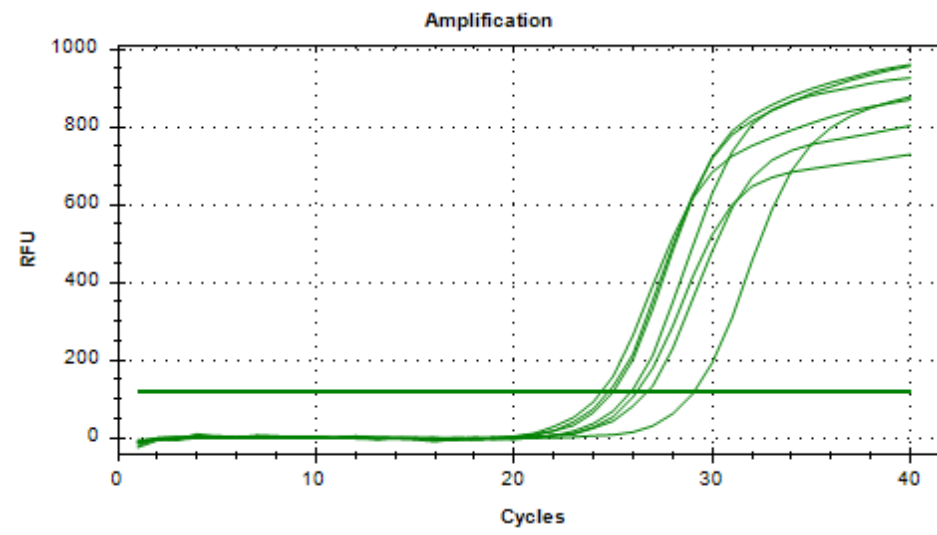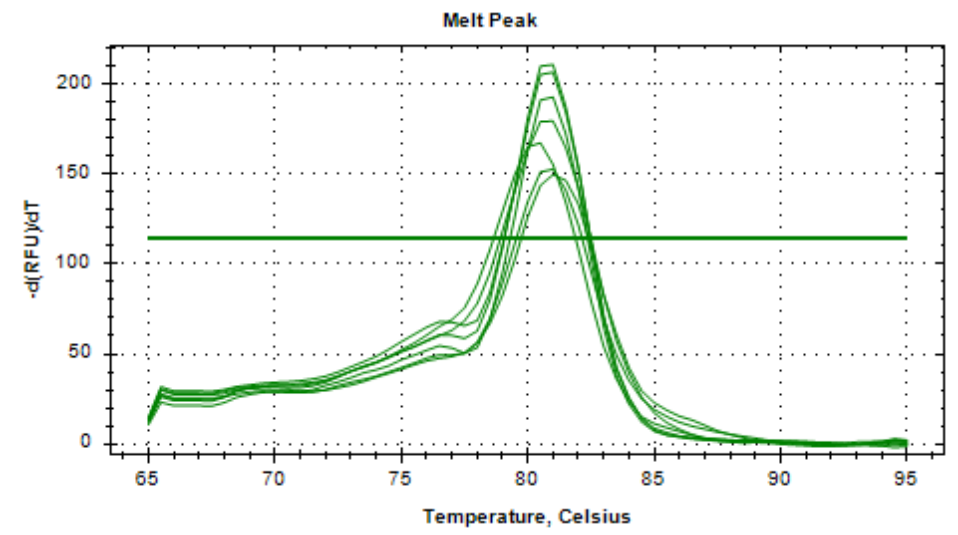

Supplement: Supplementary file 9 [file DataSheet3.PDF]

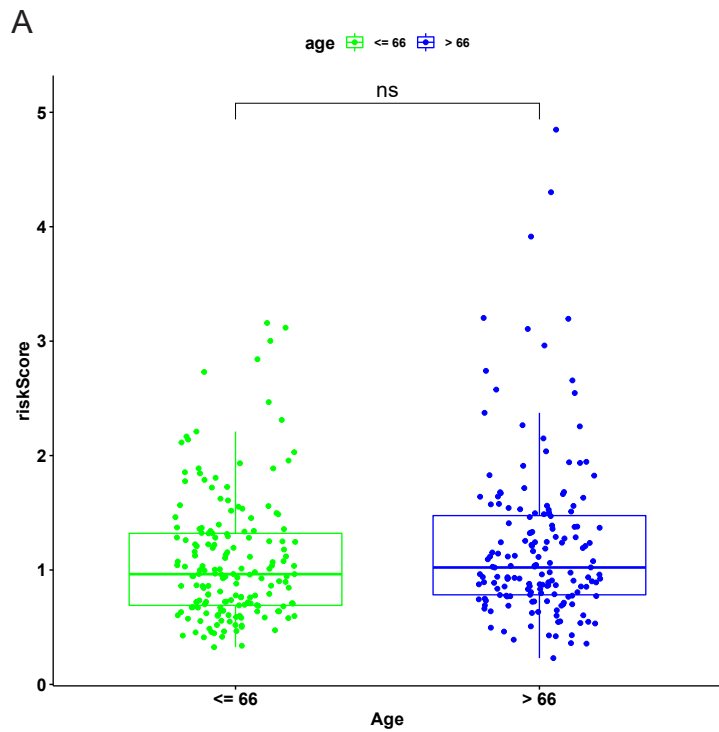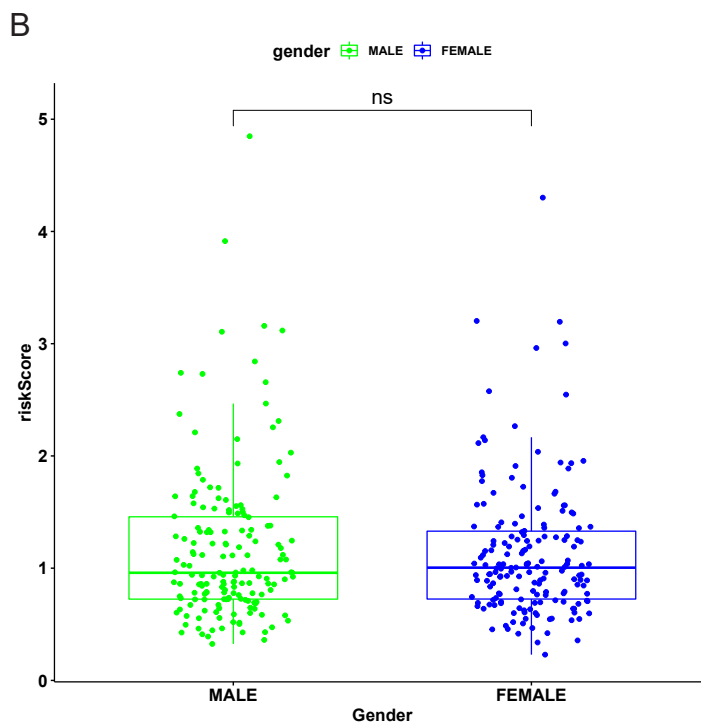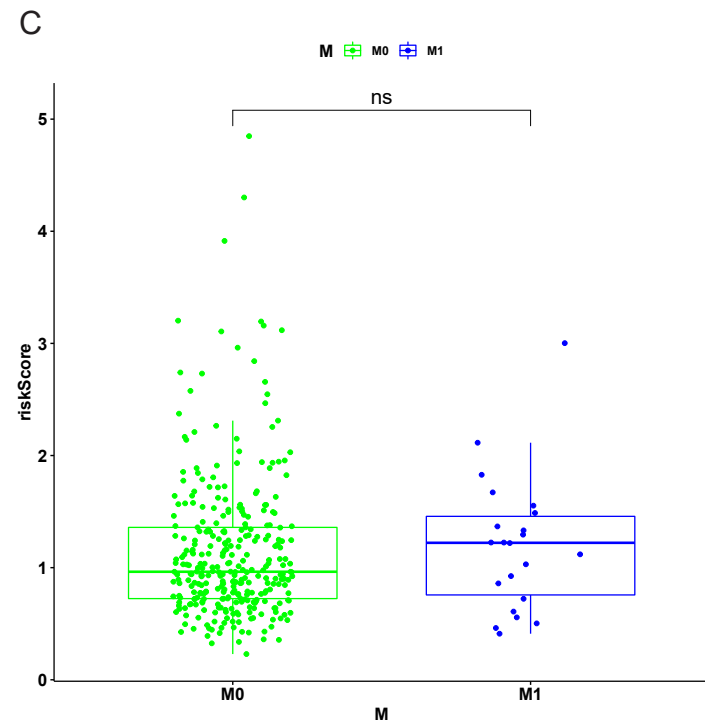

Supplement: Supplementary file 12 [file DataSheet1.PDF]
