# Supplementary material for: Construction and validation of an angiogenesis-related lncRNA prognostic model in lung adenocarcinoma
Source: Front Genet. 2023 Mar 14;14:1083593. doi: 10.3389/fgene.2023.1083593 (PMC10043447; doi:10.3389/fgene.2023.1083593)
Supplement: Supplementary file 11 [file Table2.docx]

**Clinicopathologic characteristics of 9 LUAD patients**

| Parameter | | Total  （*n=9*） |
| --- | --- | --- |
|  |  |  |
| Gender | Male | 5 (55.6%) |
|  | Female | 4 (44.4%) |
| Age(years) | ＜60 | 6 (66.7%) |
|  | ≥60 | 3 (33.3%) |
| Tumor size(cm) | ＜3 | 4 (44.4%) |
|  | ≥3 | 5 (55.6%) |
| Tumor location | Left | 2 (22.2%) |
|  | Right | 7 (77.8%) |
| Histological grade | Middle or low | 4 (44.4%) |
|  | High | 5 (55.6%) |
| Smoking history | Yes | 4 (44.4%) |
|  | No | 5 (55.6%) |
| Staging | Ⅰ | 6 (66.7%) |
|  | Ⅱ | 2 (22.2%) |
|  | Ⅲ | 1 (11.1%) |
